# Cholinergic manipulations affect sensory responses but not attentional enhancement in macaque MT

**DOI:** 10.1186/s12915-021-00993-7

**Published:** 2021-03-16

**Authors:** Vera Katharina Veith, Cliodhna Quigley, Stefan Treue

**Affiliations:** 1 Cognitive Neuroscience Laboratory, German Primate Center – Leibniz Institute for Primate Research, Goettingen, Germany; 2 Konrad Lorenz Institute of Ethology, Department of Interdisciplinary Life Sciences, University of Veterinary Medicine Vienna, Vienna, Austria; 3 Faculty for Biology and Psychology, University of Goettingen, Goettingen, Germany; 4 Leibniz ScienceCampus Primate Cognition, Goettingen, Germany

## Abstract

**Background:**

Attentional modulation in the visual cortex of primates is characterized by multiplicative changes of sensory responses with changes in the attentional state of the animal. The cholinergic system has been linked to such gain changes in V1. Here, we aim to determine if a similar link exists in macaque area MT. While rhesus monkeys performed a top-down spatial attention task, we locally injected a cholinergic agonist or antagonist and recorded single-cell activity.

**Results:**

Although we confirmed cholinergic influences on sensory responses, there was no additional cholinergic effect on the attentional gain changes. Neither a muscarinic blockage nor a local increase in acetylcholine led to a significant change in the magnitude of spatial attention effects on firing rates.

**Conclusions:**

This suggests that the cellular mechanisms of attentional modulation in the extrastriate cortex cannot be directly inferred from those in the primary visual cortex.

**Supplementary Information:**

The online version contains supplementary material available at 10.1186/s12915-021-00993-7.

## Background

Attention is a core aspect of visual information processing which induces an increased representation of attended stimuli on the neuronal level [45] in a fashion consistent across the different areas of the visual cortex [35, 37]. Because of its pervasiveness, it is important to understand its cellular and network mechanisms.

An overall key feature of attention is that it works through gain changes [30, 44]. Such gain changes have been extensively investigated and modeled in the context of gain control mechanisms prevalent in the cortex [5, 10, 25, 27, 36, 44]. Much less focus has been put on the question whether such multiplicative changes to cellular responsiveness reflect specific neurotransmitter changes (see [42] for a review). Nevertheless, there is ample evidence in favor of an involvement of the cholinergic system in attentional modulation, primarily gained from rodent studies ([17, 32, 40]; reviewed in [41]). A study performed in macaque primary visual cortex (V1) was the first in non-human primates to shed light on the underlying cellular mechanisms of the effects of spatial attention on firing rates [20]. A cholinergic agonist (acetylcholine) or antagonist (scopolamine or mecamylamine) was iontophoretically injected in the direct vicinity of the recording electrode while the monkey performed a spatial attention task. Injecting acetylcholine caused an increase in the attentional modulation of neuronal responses, while blocking the action of muscarinic, but not nicotinic, cholinergic receptors reduced attentional modulation. However, it is unclear whether V1 can serve as a model for the extrastriate cortex, as it has specific anatomical characteristics that distinguish it from other visual areas, such as a reduced amount of inhibitory neurons as well as a difference in quantity of cholinergic receptor subtypes [9, 14].

**Fig. 1:**
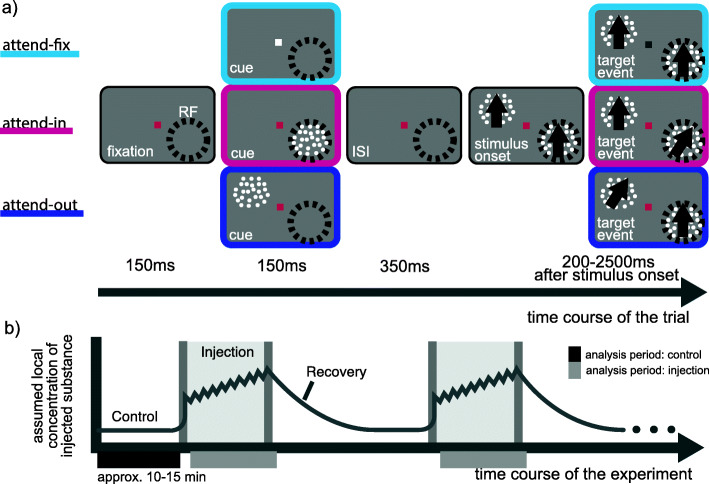
**a** Schematic trial structure of the spatial attention task. The three attention conditions, attend-fix, attend-in, and attend-out, were shown in random order. In the attend-fix trials, the monkey had to respond to a luminance change of the centrally presented fixation point. In attend-in and attend-out trials, the monkey had to respond to a direction change of the cued dot pattern. ISI inter-stimulus interval. **b** Experimental design. One full cycle of the main experiment consists of control, injection, and recovery blocks. Injection and recovery could be performed several times during one experiment. The black horizontal bar depicts the assignment of trials to the control block. The light gray horizontal bar depicts the assignment of trials to the injection block. The depicted concentration of the substance is speculative

Here, we investigated cholinergic influences on attentional modulation in the extrastriate cortex in two rhesus monkeys while they performed a spatial attention task (Fig. 1). Single-cell neuronal activity was recorded in area MT, a mid-level visual area along the dorsal visual pathway, well understood in terms of its sensory properties [4] and extensively characterized in terms of response modulation by spatial (e.g., [15, 16, 24, 45, 51]), feature-based (e.g., [33, 44]), and object-based attention (e.g., [23, 49]). Gain changes were quantified by comparing firing rates when the stimulus in the receptive field was attended vs. unattended. During recordings, we used pressure injection to pharmacologically manipulate the direct vicinity of the recorded neuron [47]. During a given recording session, either the antagonist scopolamine was used to block the muscarinic cholinergic receptor subtype or the agonist acetylcholine to enhance cholinergic action. This design allowed a within-cell comparison of attentional modulation during injection with a pre-injection baseline, for each substance separately.

While our neuropharmacological manipulation caused firing rate changes at many injection sites, we did not observe a specific effect on the attentional modulation of firing rates in MT beyond the general effect on responsivity of neurons.

Our findings show that the local cholinergic contribution to attentional effects differs across visual cortical areas, even in the same species. Therefore, V1 appears ill-suited to serve as a prototypical area for the neuropharmacological basis of attentional modulation across the visual cortex.

## Results

In this study, we aim to determine whether the cholinergic system is causally involved in the response modulation elicited by spatial attention in macaque extrastriate visual area MT.

To assess the influence of the cholinergic system on attentional modulation, we compared the neuronal responses when a stimulus in the receptive field was attended vs. unattended for two neuropharmacological conditions. In one condition, we applied a cholinergic antagonist or an agonist for one block of trials (injection block), and in the other condition, nothing was injected (control block).

**Fig. 2:**
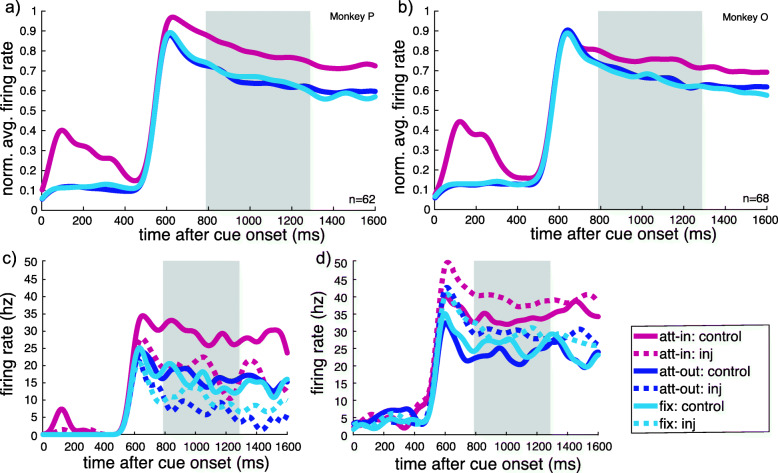
**a** Effect of spatial attention on neuronal firing rate across trial time course for monkey P (average AMI 0.094, i.e., 20.7%). Peristimulus time histograms were calculated for the control block for the scopolamine cell population of each monkey separately for the three attentional conditions. The analysis period (used to determine the AMI) is defined as 300–800 ms after RDP onset (gray shaded area). The initial firing rate modulation in the attend-in condition is due to the cue, which was present in the receptive field only during the attend-in condition. **b** Effect of spatial attention on neuronal firing rate across trial time course for monkey O (average AMI 0.047, i.e., 9.9%). **c**, **d** Effect of spatial attention on example MT cells (**c** s1-pie-035-01+01_2a, **d** s3-pie-020-01+02_1a), both of which showed a significant effect of scopolamine injection on firing rates in the fixation condition

To determine the neuronal responses across attentional conditions, we calculated the peristimulus time histograms (PSTHs) for the scopolamine cell population (monkey P, 62: monkey O, 68) during the control block, where no substance was injected. As illustrated in Fig. 2, both monkeys show clear attentional enhancement of their firing rate, as the firing rates for the attend-in condition are clearly elevated compared to firing rates in the attend-out or attend-fix conditions (red vs. blue and light blue lines).

We quantified the effect of attention on neuronal firing rate by calculating the attentional modulation index (AMI), from the relative difference of firing rate between attend-in and attend-out conditions for an analysis window 300–800 ms after RDP onset (gray-shaded areas in Fig. 2). Monkey P shows an average attentional response enhancement of the attended stimulus response over the response to the unattended stimulus of 20.7%, and monkey O shows a 9.9% attentional enhancement. As the attentional modulation is not significantly different between the two monkeys (*p* = 0.086, *U* = 4430), we pooled the data for subsequent analyses. Example single-cell PSTHs are shown in Fig. 2c, d.

**Fig. 3:**
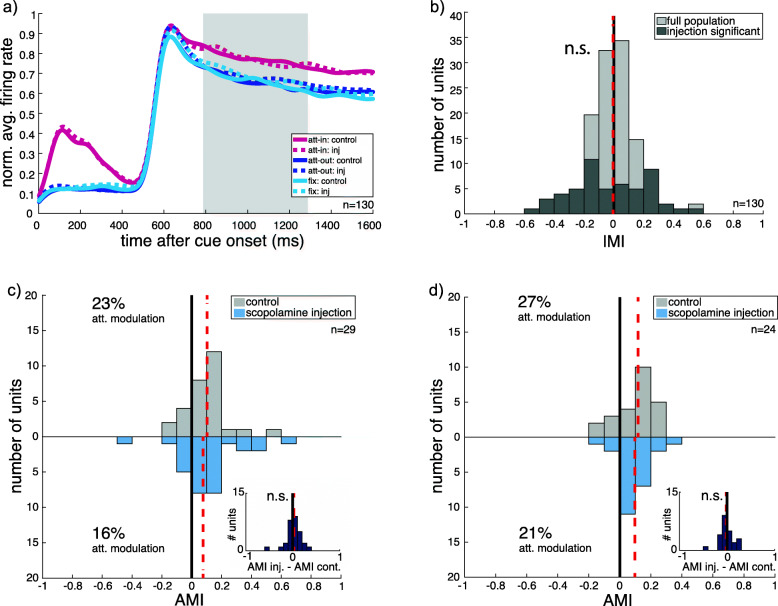
**a** PSTH of full scopolamine population of two monkeys showing neuronal spiking dynamics for the three attentional conditions during control block (solid lines, composed of the data shown in Fig. 2a and b) and injection block (dashed lines). **b** Distribution of injection modulation indices (IMI) for the sensory condition (attend-fix). Light gray depicts the full population fulfilling the first inclusion criterion, and dark gray depicts the subpopulation of cells with significant influence of injection on their firing rate. The red vertical dashed line indicates the median injection modulation for the full population. Shown data contains neuronal responses of single units from two monkeys for the preferred stimulus only. **c**, **d** Histogram of attentional modulation index for control (upper histograms) and scopolamine injection blocks (lower histograms) for cells showing a significant decrease (**c**) or increase (**d**) in firing rate at the single-cell level due to scopolamine injection. Paired differences are illustrated in insets. Red vertical dashed lines indicate the median change of the population

We next investigated the influence of the antagonist scopolamine on firing rates, shown in Fig. 3. Therefore, we compared firing rates recorded from 130 cells during the control block (solid line) with the injection block (dashed line), depicted in Fig. 3a. The injection modulation index (IMI) contrasts the control and injection block firing rates for each neuron. On average, scopolamine had no significant influence on firing rates in the sensory control condition (attend-fix, light gray histogram in Fig. 3b), showing a median IMI of − 0.005, which is equivalent to a percentage change of − 0.954% (vertical red dashed line; *N* = 130, *p* = 0.822, *W* = 4160).

As several neurons show an IMI close to zero (see Fig. 3b, light gray), it is unclear whether the injected substance reached the recorded neuron in those cases. In order to be able to investigate the influence of the substance on attentional modulation of the recorded neuron more closely, we selected only those neurons that showed a significant influence of injection on the firing rate during the fixation condition (see Fig. 3b, dark gray). Fifty-three out of 130 recorded neurons showed a significant influence by scopolamine at the single-cell level.

As scopolamine is a non-specific cholinergic antagonist, it binds to all types of muscarinic receptor subtypes. Muscarinic receptor types are known to elicit heterogeneous effects (inhibitory or excitatory) based on their variation in location and molecular composition [50]. We also observed this heterogeneity in our subpopulation as some cells showed an increase in firing rate (*N* = 24) with scopolamine injection, and some showed a decrease (*N* = 29).

### Muscarinic antagonist (scopolamine) effects on attentional modulation

As we observed diverging effects of scopolamine on firing rates, we continued by separately analyzing the subgroups defined by whether scopolamine increased or decreased the firing rate during fixation trials. The subgroup showing a decrease in firing rate with injection (Fig. 3c) was significantly influenced by spatial attention during the control block, showing a median attentional modulation of 23% (*N* = 29, *p* = 0.0003, *W* = 377). During the block of scopolamine injection, the median attentional modulation was 16.4% and remained significantly greater than zero (*N* = 29, *p* = 0.001, *W* = 360). A paired test of the AMIs during control and injection blocks did not show a significant influence of scopolamine injection on the strength of attentional modulation (*N* = 29, *p* = 0.381, *W* = 259; depicted as paired differences in Fig. 3c inset).

The subgroup showing an increase in firing rate with injection (Fig. 3d) also showed a significant effect of attention with a median firing rate modulation of 26.6% (*N* = 24, *p* = 0.0009, *W* = 261). When scopolamine was injected, a median attentional modulation of 21.4% was observed, again significantly different to zero (*N* = 24, *p* = 0.0002, *W* = 270). The AMI comparison between control and injection blocks did not reach significance (*N* = 24, *p* = 0.833, *W* = 142; depicted as paired differences in Fig. 3d inset).

To summarize the effects of local scopolamine injection, we can report that on average, local application of scopolamine led to significant changes in firing rate during the sensory control condition in around 40% of the recorded neurons, with roughly equal proportions of increased and decreased firing rate. Both subgroups of SUs showed significant effects of attention on firing rate. However, we did not detect any systematic change in the magnitude of attentional enhancement during scopolamine injections. We repeated all analyses using only SUs from sessions with 0.1 M scopolamine (*N* = 73, 38 of which were significantly influenced by injection, 21 with decreased and 17 with increased firing rates) and the pattern of results was the same.

### Cholinergic agonist (acetylcholine) effects on attentional modulation

**Fig. 4:**
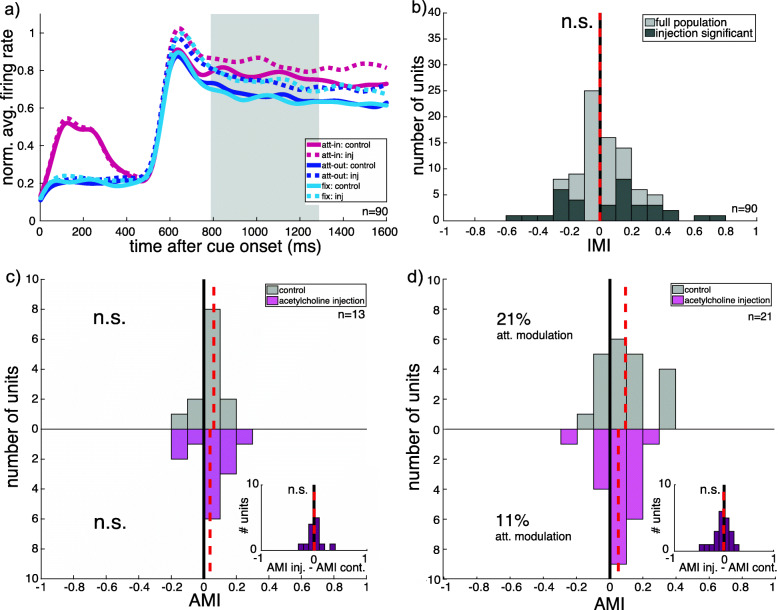
**a** PSTH of full acetylcholine population of two monkeys showing neuronal spiking dynamics for the three attentional conditions (attend-in: red, attend-out: blue, fixation: light blue) during control block (solid line) and injection block (dashed line). **b** Distribution of injection modulation indices (IMI) for the sensory condition. Light gray depicts the full population fulfilling the first inclusion criteria, and dark gray depicts a subpopulation of cells with significant influence of injection on their firing rate during the fixation condition. Red vertical dashed line indicates median injection modulation for the full population. Shown data is derived from neuronal responses of single units from two monkeys for the preferred stimulus only. **c**, **d** Distribution of attentional modulation index (AMI) for control and acetylcholine injection block of the significantly affected cells, showing a decrease (**c**) or an increase (**d**) in firing rate. Red vertical dashed lines indicate median attentional modulation

In order to investigate a general cholinergic involvement in attentional modulation, we injected acetylcholine in the direct vicinity of the recorded neuron. We performed the same analysis for this substance as for the muscarinic antagonist scopolamine. Again, we compared firing rates during the control block (Fig. 4a; solid lines) and acetylcholine injection block (dashed lines) using the IMI.

Figure 4b illustrates the distribution of injection modulation index for the full population of cells (light gray) and for the subpopulation showing a significant influence of acetylcholine injection on firing rate during the sensory control fixation condition (dark gray). The median IMI does not significantly differ from 0 for the full population of cells exposed to acetylcholine injection (*N* = 90, *p* = 0.39, *W* = 2263). As observed for the scopolamine subpopulation, the subpopulation of cells significantly affected by acetylcholine (34 of 90 SUs, approximately 38%) shows a heterogeneity in their response: As illustrated in Fig. 4, 13 cells show a decrease (c) and 21 cells an increase (d) in firing rate due to injection during the sensory control condition.

During the control block, where no substance is injected, the subgroup of cells with increased firing rate during acetylcholine injection (d) shows a median attention modulation of 20.8% and is significantly influenced by spatial attention (*N* = 21, *p* = 0.01, *W* = 187). During injection, the median attentional modulation was 10.6%, still significantly different from zero (*N* = 21, *p* = 0.007, *W* = 191). This reduction is not significant (*N* = 21, *p* = 0.43, *W* = 92).

The subgroup showing a decrease (c) in firing rate does not show a significant effect of spatial attention (*N* = 13, *p* = 0.11, *W* = 69). When acetylcholine was injected, attentional modulation was 8.12% (*N* = 13, *p* = 0.19, *W* = 65). Acetylcholine injection has no effect on the magnitude of spatial attention modulation (*N* = 13, *p* = 0.95, *W* = 47). In summary, local acetylcholine injection does not affect the average attentional enhancement. Even when we select only those cells that are significantly influenced by the injected substance, we find no evidence for an involvement of cholinergic receptors in modulating spatial attention in area MT.

## Discussion

The aim of this study was to establish whether the mechanism of attentional modulation in the extrastriate cortex leverages a specific neurotransmitter system. As evidence has already been found in the striate cortex [20] for a role of acetylcholine, we focused on the effect of the local cholinergic intervention. Acetylcholine elicits a multiplicative increase in neuronal gain in areas V1 [12] and the medial temporal area MT in anesthetized macaques [43] and has been suggested as a main regulatory neurotransmitter of selective attention [26].

Here, we focused on the modulation of sensory responses by spatial attention in MT of the extrastriate cortex in awake behaving macaques. MT is a core component of the dorsal visual pathway, and attentional modulation of its direction-selective responses to visual motion has been well-characterized [4, 44, 46, 49]. Modulating the cholinergic system in MT, we observed clear effects on sensory responses, but no specific modulation of attentional effects.

### Effectiveness of local pharmacological interventions in influencing sensory responses

Based on the results in V1 [20] for the muscarinic antagonist scopolamine, we first determined the effect of scopolamine injections on firing rates of our population of MT neurons. In contrast to the results in V1, on average, we did not observe a change in firing rate between injection and control blocks. As scopolamine is a non-specific muscarinic antagonist, it binds to all subtypes of muscarinic acetycholine receptors, which are known to elicit heterogeneous (inhibitory or excitatory) effects due to variations in receptor location and molecular composition [50]. When we focused on those cells significantly affected by scopolamine in the sensory control condition (40%), we confirmed this heterogeneity: some cells showed an increase in firing rate with scopolamine injection (45%), and some showed a decrease (55%). This result is in line with anatomical results showing that the muscarinic receptor type m1, which is proposed to be involved in attentional modulation, is located on inhibitory and excitatory neurons in area MT [11].

In the recording sessions where we increased the concentration of the neuromodulator acetylcholine in the local circuitry, we again found heterogeneous effects, as some neurons showed an increase (60%) and some a decrease (40%) in firing rate. Cholinergic injections can lead to an inhibitory tone [11], and an increase in GABA release with iontophoretic acetylcholine injection has been shown in area V1 [13]. In contrast, cholinergic injections might increase firing rates, because the release of acetylcholine has been shown to induce an increased release of glutamate (the brain’s main excitatory neurotransmitter) or when recording from GABA neurons that are excited by acetylcholine [19]. In line with these observations from V1, a study in area MT of anesthetized rhesus monkeys also reports heterogeneous effects [43].

### Muscarinic receptor contribution to attentional modulation

Having established the effectiveness of our cholinergic pressure injections, we turned to the core question of our study by comparing the magnitude of attentional modulation with and without injections. Although we observed a clear attentional modulation in our population of neurons and a modulation of firing rates by scopolamine injections, we did not observe a specific effect of scopolamine on the attentional modulation, even when restricting the analysis to the highest drug concentration used. In spite of the clear effect of injection on firing rates in a subset of the recorded cells, neither cell subgroup showed a change in the magnitude of attentional modulation during injection, which argues against a muscarinic involvement in the modulation of sensory responses by spatial attention in extrastriate cortex.

In the primary visual cortex (V1), scopolamine reduces attentional modulation [20]. This might either reflect a direct consequence of blocking muscarinic receptors, which are predominantly found on inhibitory cells in V1 [14] or could be the result of subsequent changes in the local cortical microcircuit. As spatial attention effects on neuronal responses are thought to increase in magnitude with every successive step of the visual hierarchy [29], a reduction of attentional influences should be more easily observed in area MT compared to area V1. However, we did not observe this pattern of results. Although we indeed observed a strong influence of spatial attention on the neuronal firing rate, the muscarinic antagonist scopolamine did not significantly influence attentional modulation.

### Acetylcholine effects on attentional modulation

The basal forebrain is considered to be the main cholinergic source providing all cortical areas with acetylcholine via topographically organized but rather broad innervations [3, 18, 22]. In top-down processes such as sustained attention, the cholinergic supply is thought to be regulated via direct connections from prefrontal areas, either to the basal forebrain or to posterior attention systems [39]. It has been proposed that variations in the local circuits in the sensory cortex enable spatially and temporally focused cholinergic effects [8, 21, 28, 48], in line with the requirements of a dynamic attentional system. Despite these findings, we did not find a specific effect of acetylcholine injections on the magnitude of attentional modulation even for the subset of cells showing significant effects of acetylcholine in the sensory control condition.

Our experimental design is similar to the one used in a previous study in macaque primary visual cortex [20]. Both studies chose stimuli that optimally drive cells in the visual area under examination, employed a task to manipulate the spatial focus of sustained selective attention, manipulated the local cholinergic milieu using scopolamine and acetylcholine, and recorded predominantly from close to the soma (given the signal-to-noise ratio there). Given the larger distance between injection and recording sites used in our study, this means we maximally affected receptors located further away from the soma compared to the experiment performed in V1 by Herrero et al. Although both studies observed clear effects on firing rate in comparable proportions of recorded cells, the difference in injection location could have caused different attention effects. However, we did not find any apparent effect of substance concentration, volume injected, or inter-tip distance on the pattern of effects on firing rate (see Additional file 1: Figures S4 and S5). Alternatively, the difference between the two studies might reflect a heterogeneity of cholinergic system actions across the visual cortex [7], difference in which cell types are more readily detectable given differences in electrode parameters, differences in proportions of cell types between areas [14], or a difference in the cortical layers reached due to anatomical orientation of different brain areas.

The antagonist and agonist used in this study are known for their broad binding characteristics. This has led to their use as standard substances for investigating the general roles of the cholinergic system on a cellular level as well as in cognitive tasks [1, 20]. More specific antagonists, binding to just one type of receptor, need to be used for more fine-grained analyses*.* Of highest interest would be the muscarinic subtype m1, as it is proposed to be involved in attentional modulation and is found on both inhibitory and excitatory neurons in macaque MT [11].

## Conclusions

In summary, while our study clearly documents cholinergic influences on sensory responses in macaque area MT, we did not find an additional cholinergic effect on attentional gain changes. Neither a muscarinic blockage nor a local increase in acetylcholine led to a significant change in the magnitude of spatial attention effects on firing rates. This suggests that the mechanisms of attentional modulation in the extrastriate cortex at the cellular level are different from those in the primary visual cortex, where the cholinergic system has previously been shown to specifically modulate attentional effects.

Our observations are in line with the suggestion that the cholinergic system plays a central role in the gain control circuitry of the visual cortex [41]. The lack of an additional specific role in attentional modulation on the other hand challenges the close coupling that has been suggested between the gain control circuitry and the mechanisms of attentional modulation.

## Methods

### Animal welfare

The scientists in this study are aware and are committed to the great responsibility they have in ensuring the best possible science with the least possible harm to any animals used in scientific research [38]. Details about our animal care and handling have been reported previously [25, 51]. We summarize relevant details here: The animals were group-housed with other macaque monkeys in facilities of the German Primate Center in Goettingen, Germany, in accordance with all applicable German and European regulations. The facility provides the animals with an enriched environment including a multitude of toys and wooden structures [2, 6], natural as well as artificial light, exceeding the size requirements of the European regulations, including access to outdoor space. Surgeries were performed aseptically under balanced anesthesia using standard techniques, including appropriate peri-surgical analgesia and monitoring to minimize potential suffering. The German Primate Center has several staff veterinarians that regularly monitor and examine the animals and consult on procedures. During the study, the animals had unrestricted access to food and fluid, except on the days where data were collected or the animals were trained on the behavioral paradigm. On these days, the animals were allowed unlimited access to fluid through their performance in the behavioral paradigm. Here, the animals received fluid rewards for every correctly performed trial. Throughout the study, the animals’ psychological and veterinary welfare was monitored by the veterinarians, the animal facility staff, and the lab’s scientists, all specialized in working with non-human primates.

We have established a comprehensive set of measures to ensure that the severity of our experimental procedures falls into the category of mild to moderate, according to the severity categorization of Annex VIII of the European Union’s directive 2010/63/EU on the protection of animals used for scientific purposes (see also [34]).

### Monkey surgery

Two male macaque monkeys (*Macaca mulatta*) were implanted with custom-made titanium head holders to prevent head movement during the recording and gaze tracking. Additionally, they were implanted with a recording chamber (Crist Instruments, Hagerstown, MD, USA, or 3DI, Jena, Germany), first over one and subsequently over the other hemisphere. The position of the recording chambers was planned based on anatomical fMRI scans using a MATLAB-based (The MathWorks, Inc., Natick, MA) software [31].

### Apparatus

During recordings, monkeys were seated in a custom-made primate chair in a dark cabin at a distance of 57 cm from the computer monitor (Quato Display 240 m). Visual stimuli were presented with a refresh rate of 60 Hz and a resolution of 1920 × 1200 pixel. Eye position was recorded using an eyetracker (ET49, Thomas Recording, Giessen, Germany) with an acquisition rate of 230 Hz. Acute recording/injection was performed using a multielectrode manipulator equipped with a pressure injection system and containing two recording electrodes and an injection micropipette (MiniMatrix, Thomas Recording). The majority of recordings had inter-tip distance of electrodes and micropipette of approx. 300 μm. Later recordings were performed using an adaptor to achieve a closer spacing of approx. 100 μm, bringing us closer to the spacing used by Herrero et al. [20], which was in the order of 10s of μm. In detail, we used this adaptor in one of the monkeys for both substances, acetylcholine and scopolamine, in 15% and 30% of the cells respectively. Additional file 1: T1 provides a detailed list of the distances used for every recorded neuron. For further details about preparation, handling, and care of the system, see Veith et al. [47]. Recording of neuronal signals and real-time spike sorting was performed with a data acquisition system (MAP, Plexon Inc., Dallas, USA). All stimuli were generated and presented using custom-made software built for real-time visual experiments running on an Apple Macintosh PowerPC. In addition, the software monitored eye position, controlled fluid reward release, and collected behavioral as well as electrophysiological data.

### Stimuli

The stimuli used were random dot patterns (RDPs) presented on a uniform gray background (luminance 27 cd/m^2^). Each RDP consisted of small bright dots (size 0.1 deg, luminance 38 cd/m^2^, Weber luminance contrast 40%), coherently moving linearly within a circular, stationary aperture. One RDP was placed at the most responsive part of the receptive field (RF), and the second RDP was placed diametrically opposite, relative to the centrally presented fixation point. The speed of the moving dots was adjusted to the recorded cell’s preference within a range of 4–12 deg/s, and the preferred movement direction of the recorded cell was defined based on a pre-experiment tuning session. The radius of the RDP was increased when stimuli had to be placed very eccentrically in order for the stimulus change (see below) to remain detectable for the monkey (range of 2–3 deg).

### Substances

In order to manipulate the cholinergic system, we used the general agonist acetylcholine and the muscarinic antagonist scopolamine in various concentrations and volumes (Sigma-Aldrich, St. Louis, MO, USA). All substances were diluted in sterile 0.9% (0.154 mol/l) saline (NaCl, BBraun, Melsungen, Germany). For details, see Veith et al. [47].

For acetylcholine, concentrations of 0.1, 0.15, and 0.2 mol/l and injection rates of 2, 3, and 4 nl/min were used. Scopolamine was injected with concentrations of 0.01, 0.05, and 0.1 mol/l and rates of 1, 2, and 4 nl/min. As a control, saline was injected with a volume of 2, 4, and 6 nl/min in order to mimic the influence of different volumes onto the recorded neuron. Additional file 1: T1 provides a detailed list of the concentration and total injected volume combinations used.

Measured pH values stayed at approx. 5 for all substances used. Different concentrations of the substances used had only weak influences on pH values.

### Behavioral task

This task was designed to guide the monkeys’ sustained selective spatial attention to various locations on the monitor. Therefore, the two monkeys were trained to detect a motion direction change in the RDP that was cued at the beginning of the trial. The cue was either placed within the neuron’s receptive field (attend-in) or in the other hemifield (attend-out). In the control (sensory) condition, both RDPs were task irrelevant (attend-fix).

Monkeys initiated each trial by holding a lever and fixating the centrally presented fixation point. In the attend-in and attend-out conditions, the centrally presented fixation point remained red (square with side length 0.16 deg; luminance 14 cd/m^2^) during the entire trial. After a delay of 150 ms, a static dot pattern, serving as an exogenous cue, was presented for 150 ms at the future stimulus position either within the neuron’s receptive field (attend-in) or outside of it (attend-out). An inter-stimulus interval of 350 ms followed, where only the fixation point was shown. Subsequently, two RDPs were presented on the screen, one placed within the neuron’s receptive field and the other in the opposite hemifield. Both moved linearly in the same direction, either the preferred direction of the recorded neuron or its null direction (preferred direction + 180 deg). Monkeys had to respond to a slight direction change (duration 130 ms) at the cued location (target) and had to ignore a direction change at the uncued location (distractor). The angle of direction change varied from 25 to 35 deg, depending on the stimulus eccentricity, and remained the same within a recording session. The aim was to adjust the task difficulty depending on stimulus position, choosing bigger angles for more eccentric stimuli. The direction change could occur in a time window of 200–2500 ms after stimulus onset, to ensure that spatial attention was maintained at the cued location. In 1/10th of the trials, no direction change happened at the target position and monkeys were rewarded for not responding until the trial ended.

In the sensory condition (attend-fix), the monkeys had to respond to a slight luminance change of the fixation point and had to ignore direction changes of the two shown RDPs. Here, the fixation point changed from red to gray immediately after the trial began. The behaviorally relevant luminance change (from 85 to 52 cd/m^2^) of the fixation point could occur in a time window of 200–2500 ms after the fixation point color change. Additionally, we had a baseline condition, where only the fixation point was shown on the screen and the monkeys again had to detect a luminance change.

In all conditions, monkeys had to respond by releasing a lever within a fixed time window after target onset and were rewarded with a drop of juice. Trials of all attentional and sensory conditions were presented in random order. A trial was aborted if eye fixation was interrupted or eye gaze moved outside of the fixation window (1.2 deg radius around the fixation spot). Aborted trials were repeated later to allow comparable number of trials per condition.

### Experimental procedure

Single unit activity was recorded using two single tungsten electrodes of two different impedances (0.2–0.5 MΩ and 1–2 MΩ) placed in a multielectrode recording system (Thomas Recording, Giessen, Germany). Data was filtered (150 Hz–5 kHz) and amplified (gain range 1000–32,000). Isolation of single units was performed using online window discrimination (RASPUTIN, Plexon Inc., Dallas, TX, USA), and later offline sorted (Plexon Offline Sorter version 3.3.5).

### Characterization of isolated cells

In order to confirm isolated cells were in area MT, we examined their response properties using a mapping and tuning experiment at the start of each recording session. The mapping experiment determined RF extent and the region at which visual stimulation elicited the highest response. This was done by manually moving a static dot pattern on the monitor using mouse control. If the RF size matched the size of an average MT cell (diameter approx. 5 deg, eccentricity dependent), we continued with the tuning task in which one RDP was placed in the RF. While the monkeys performed a luminance detection task, where a slight luminance change of the centrally presented fixation point had to be detected, the dots of the RDP performed brief coherent linear movements in a pair of opposing directions (e.g., 0/180) at various speeds (2, 4, 8, and 12 deg/s). The direction pair differed on each trial in steps of 30 degrees, presented in random order. Based on the tuning profile of the neuron, which was computed online, units were identified as MT neurons and the preferred direction and speed were defined for the subsequent attentional task. When recording several units simultaneously, stimulus properties were chosen to optimally activate all neurons.

### Main experiment with pharmacological manipulations

With the information gained in the mapping and tuning tasks, we generated the main experimental task with stimulus properties targeting the isolated neuron(s).

The main experiment is subdivided into three, possibly repeating, blocks: control, injection, and recovery (see Fig. 1b).

During all blocks, the monkeys performed all attentional task conditions in random order. During the control block, neuronal activity was measured in the absence of pharmacological influence. After sufficiently many hit trials (at least 11 repetitions of each condition), a specific amount of a substance was injected every minute, or twice a minute, using pressure injection (gray-shaded area in Fig. 1b). In total, 2 different substances were used in this study: acetylcholine (general agonist) and scopolamine (muscarinic cholinergic antagonist). As a control, saline was injected. Only one substance was used per recording session.

A recovery block followed the injection block, in which no substance was injected. In this block, the neurons’ activity should recover, if affected during the injection block, to the same value as in the control period. In most of the experiments, one full cycle, covering control, injection, and recovery block, was performed. In other rare cases, an additional or even a third cycle of pharmacological manipulation was recorded.

### Data analysis

With our custom-made software, we were able to analyze the behavioral data online during the recording session, as well as the spike train of the isolated cells. This helped us gain an initial impression of the data quality while recording. Final data analysis was performed offline using custom scripts written in MATLAB (R2016b), after offline sorting of the recorded neural data.

For the main analysis, only the response to the preferred direction of each neuron was used. Firing rates were averaged in an analysis window of 300–800 ms after RDP onset for every trial.

We compared firing rates in the absence (initial control block) and presence of a drug (injection block/s). To determine statistical significance, two-sided non-parametric tests (Wilcoxon signed-rank for paired data or a test of one sample against a median of 0; rank-sum for comparing two independent samples) were used unless otherwise indicated.

The trials recorded before the first injection provided control data. The injection block started with the first trial occurring > 150 s after the first injection and ended 150 s after the last injection (see Fig. 1b).

### Inclusion criteria

The first inclusion criterion examined data quality for each recorded neuron, independent of the effects of injection and attention. Here, we only included data that contained enough trial repetitions during the injection block(s) for every task condition (a minimum of 3 trials). Additionally, recorded cells had to respond with a certain strength to the preferred stimulus (minimum firing rate of 7 spikes/s). A responsiveness check was also performed comparing the sensory conditions for preferred and null direction and fix-only during the control block. Cells were included in further analysis if they showed significant differences between the three fixation conditions (Kruskal-Wallis test, *p* < 0.05).

A second criterion was applied to define the subsets of cells showing a significant effect of each injected substance. We compared the distribution of firing rates in the preferred direction sensory condition (attend-fix) in the control block with those in the injection block (Wilcoxon rank-sum test, *p* < 0.05).

Only one substance was injected in each recording session. One hundred thirty well-isolated MT single units of two monkeys, recorded from both hemispheres, fulfilled the first inclusion criteria when using the muscarinic antagonist scopolamine. Twenty-eight units were used for saline control. Ninety units were used to analyze the effect of general agonist acetylcholine.

### Quantifying modulation

We measured the effects of attention and injection in a fixed time window during the sustained response of the neuron to the moving stimulus in its receptive field (300–800 ms after the onset of the RDPs). An injection modulation index (IMI) was used to quantify the influence of injection on firing rates. It was defined as the difference in average firing rate, divided by the sum.

$$ \mathrm{IMI}=\frac{R_2-{R}_1}{R_2+{R}_1} $$

where *R*_1_ is the firing rate in the control block and *R*_2_ in the injection block. Therefore, a positive IMI indicated a response enhancement due to injection. The IMI was calculated separately for all conditions: attend-fix, attend-in, and attend-out.

In order to test for injection effects on attentional modulation, an attentional modulation index (AMI) was calculated and compared for control and injection blocks.

The AMI was used to measure the effect of attention on firing rate and was defined as:

$$ \mathrm{AMI}=\frac{Q_2-{Q}_1}{Q_2+{Q}_1} $$

where *Q*_1_ is the respective firing rate in attend-out trials and *Q*_2_ in attend-in trials. Positive values indicate an increase in response by attention, whereas negative values denote suppression.

The AMI and the IMI were also used to define the percentage modulation in firing rate due to attention or injection.

$$ \mathrm{percAMI}=\frac{2\times \mathrm{AMI}}{1-\mathrm{AMI}}\times 100 $$

The percentage change for the IMI was calculated in the same manner as for the AMI.

### Test for potential injection effects

In order to exclude an influence of the injection process per se on the neuronal firing rate, we injected saline as a control substance, following the same protocol as the other injections. As depicted in Additional file 1: Figure S1, 28 of those neurons were included in the analysis (see criteria above). This group of neurons was not significantly affected by saline injection during the fixation condition (fixation condition vs. 0; *N* = 28, *p* = 0.095, *W* = 129).

Additionally, we could exclude the influence of the injection process on attentional enhancement, as attention modulation was not significantly changed with saline injection (*N* = 28, *p* = 0.479, *W* = 171, inset histogram in Additional file 1: Figure S1b).

## Supplementary Information

**Additional file 1: Figure S1.** Results from control substance NaCl. **Figure S2.** Effect of injected substance on the attentional modulation index (AMI) of pooled data significantly affected by injection. **Figure S3.** Lack of relationship between injection and attention effects. **Figure S4.** No apparent effect of scopolamine volume and concentration. **Fiure S5.** No apparent effect of acetylcholine volume and concentration or of inter-tip distance. **Table S1.** List of analyzed SUs with pharmacological parameters used. **TableS2.** AMI and IMI values for analyzed SUs.

## Data Availability

The complete modulation indices and many other parameters for every cell are provided in the Supplementary Materials. Further data and analysis code are available upon reasonable request to the corresponding author.
