# Supplementary material for: Cholinergic manipulations affect sensory responses but not attentional enhancement in macaque MT
Source: BMC Biol. 2021 Mar 16;19:49. doi: 10.1186/s12915-021-00993-7 (PMC7967954; doi:10.1186/s12915-021-00993-7)
Supplement: Supplementary file 1 — Additional file 1: Figure S1. Results from control substance NaCl. Figure S2. Effect of injected substance on the attentional modulation index (AMI) of pooled data significantly affected by injection. Figure S3. Lack of relationship between injection and attention effects. Figure S4. No apparent effect of scopolamine volume and concentration. Fiure S5. No apparent effect of acetylcholine volume and concentration or of inter-tip distance. Table S1. List of analyzed SUs with pharmacological parameters used. TableS2. AMI and IMI values for analyzed SUs. [file 12915_2021_993_MOESM1_ESM.pdf]

Supplementary Material

In this study, we report an effect on firing rate of some single units (SUs) in MT after local injection of cholinergic agents. When we examined all recorded SUs, as well as the subset of SUs significantly affected by injections, we failed to find a significant effect on the magnitude of attentional modulation of firing rates. This was also the case when we divided the significantly affected subset into SUs with increased vs. decreased firing rates. As our main finding constitutes a null effect for a cholinergic influence on attentional modulation of firing rates in area MT, we carried out some further, exploratory analyses, some of which are depicted here.

First, we provide the results of our analysis pipeline applied to recordings during control injection of saline solution (Fig S1) to show that saline had no effect on firing rates in the sensory condition (stimulus present in receptive field; contrast change task performed at fixation), on average (Fig S1A, p=0.095, W=129, n=28) and that the magnitude of attentional modulation was unchanged with injection (Fig S1B inset, p=0.479, W=171, n=28).

Second, we provide the results of our analysis pipeline applied to the subsets of SUs for which firing rates in the sensory condition were significantly affected by injection of scopolamine (Fig S2A) and acetylcholine (Fig S2B). These populations are depicted in the main text in sub-groups split by whether the firing rate was increased or decreased. Despite the larger sample sizes, there is no significant effect on the magnitude of attentional modulation.

Third, we explored whether there might be a hidden interaction between the magnitudes of attentional and injection effects, for example that those MT cells most strongly affected by top-down attention are also more strongly affected by cholinergic manipulation. No such picture of results was evident, and we provide a plot (FigS3) of the magnitude of attentional modulation without injection (AMI control) against the magnitude of injection effect in the sensory condition (IMI fixation) as an example.

We furthermore investigated the possible influence of pharmacological parameters (volume of substance injected, concentration of substance) on the size of the injection effects. Fig S4 shows the magnitude of the injection effect in the sensory condition as a function of different concentrations and volumes for scopolamine. [We additionally analysed only those SUs for which the highest concentration was used (see Results & Discussion) and found no difference in results.] FigS5 shows the same data, additionally divided by inter-tip distance, for acetylcholine. Volume, concentration, and inter-tip distance do not appear to have a systematic effect on magnitude of injection effects.

Finally, when sample size allowed, we looked at some other measures that have previously been shown to be affected by attentional state (data not shown). Initial cursory analyses of Fano factor and burstiness (inter-spike intervals) did not reveal any significant

cholinergic modulation of attentional effects and were not pursued further. In addition, we looked at waveform parameters that have previously been used to putatively assign cell types to recorded neurons. A comparison of parameters derived from SUs for which a given injected substance increased vs. decreased firing rates did not result in significant differences for any estimated waveform parameter or substance.

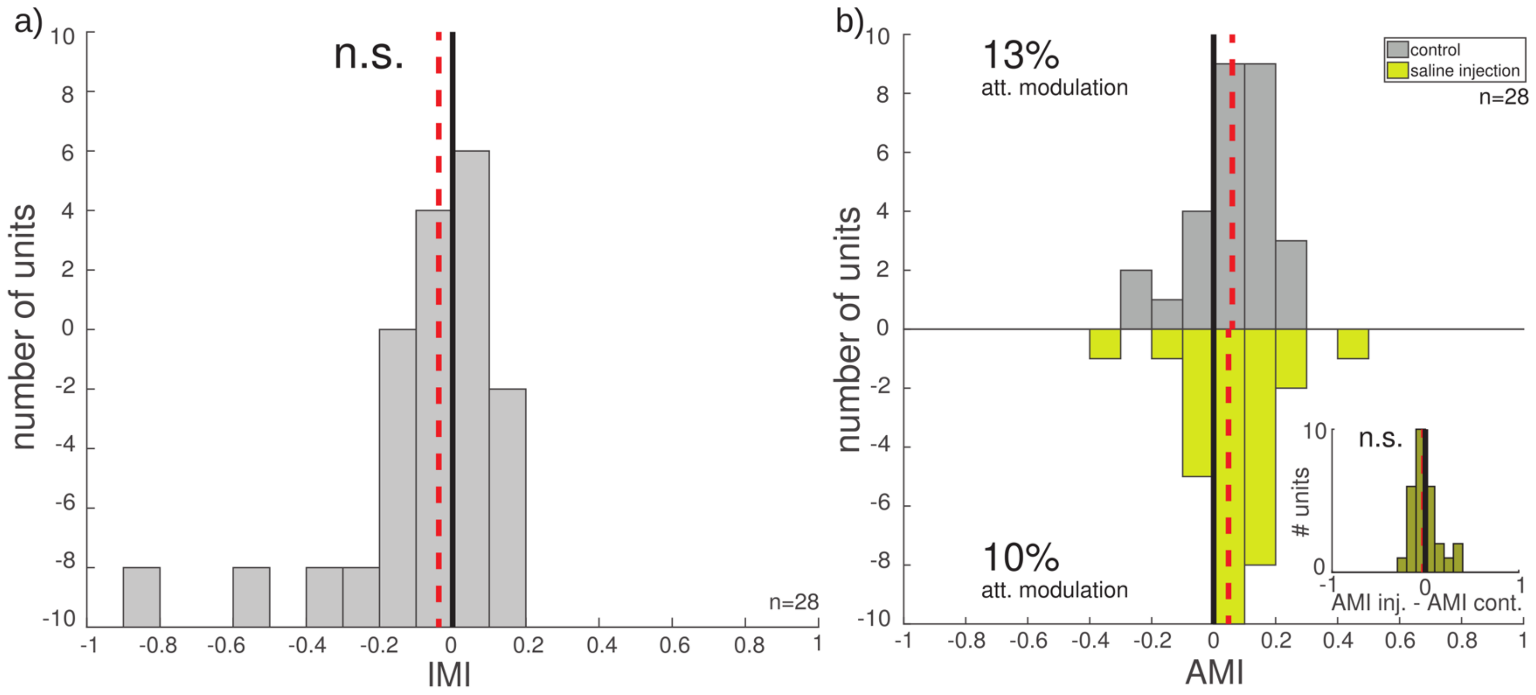

Supplementary Figure 1: Results from control substance NaCl 1. a) Distribution of saline injection modulation indices (IMI) during sensory condition. b) Distribution of attentional modulation (AMI) for the control (upper histogram) and the saline injection (lower histogram). Inset shows distribution of paired differences (injection minus control). Red vertical dashed lines indicate the median injection modulation. Shown data contains neuronal responses of single units from two monkeys for the preferred stimulus only.

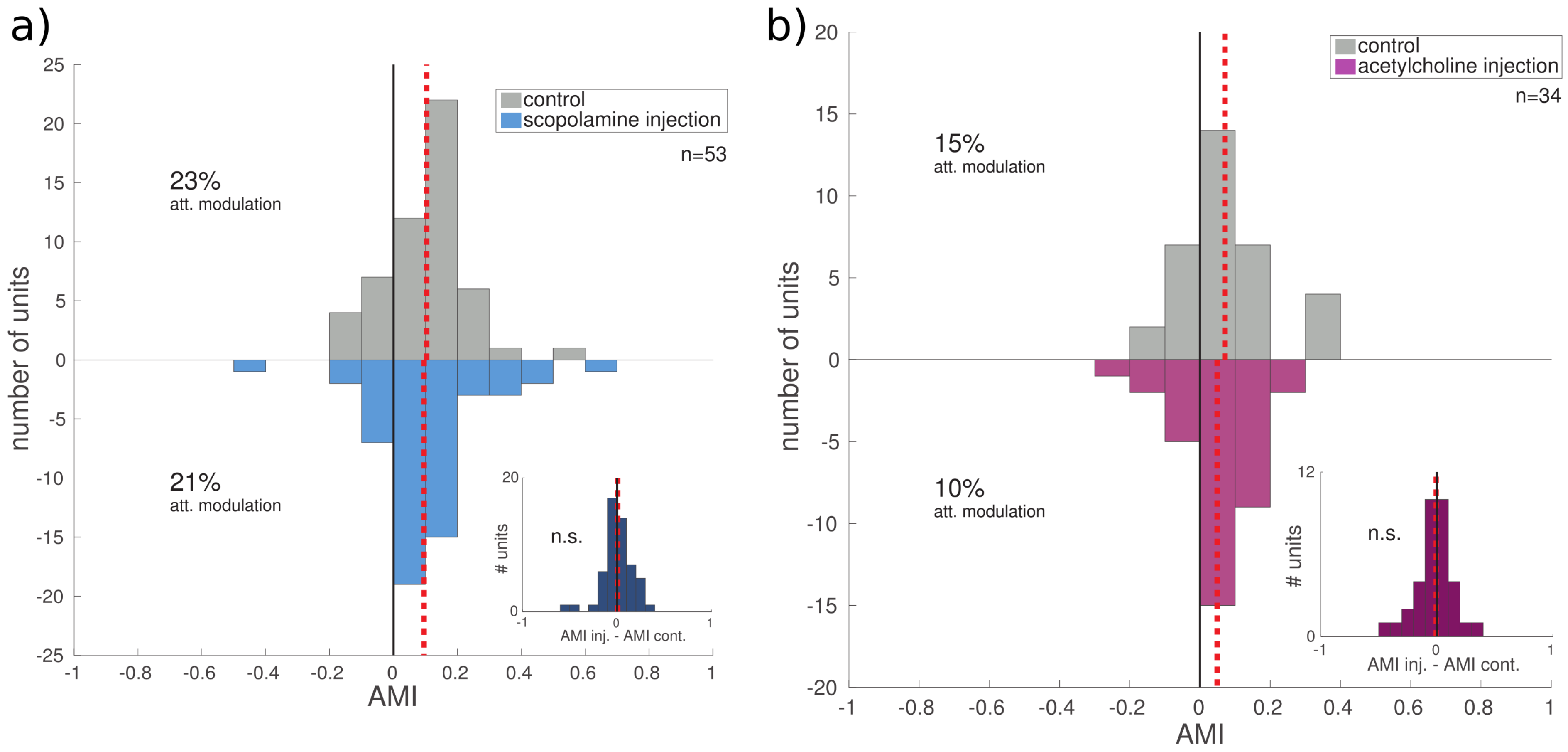

Supplementary Figure 2: Effect of injected substance on the attentional modulation index (AMI) of pooled data significantly affected by injection a) Histogram of attentional modulation index for control (upper histograms) and scopolamine injection blocks (lower histograms) for cells showing a significant effect on firing rate at the single-cell level due to scopolamine injection (pooling the data plotted as Fig 3c and d in the main text). Paired differences are illustrated in insets. Red vertical dashed lines indicate the median change of the population. b) As in panel a, but for acetylcholine injections (pooling the data plotted as Fig 4c and d in the main text).

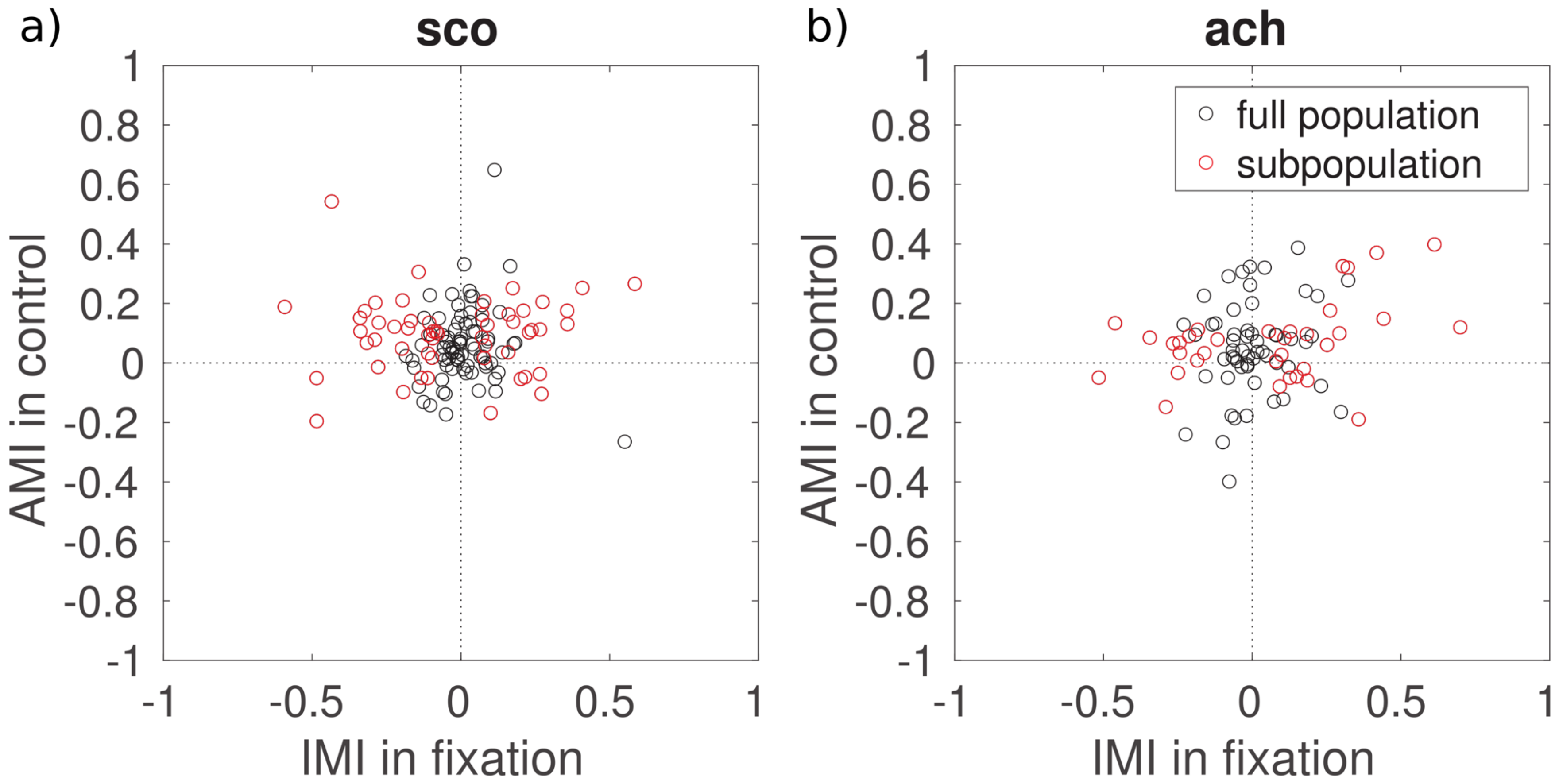

Supplementary Figure 3: Lack of relationship between injection and attention effects a) Scatter plot depicting the size of the attention effect before injection (y-axis) and the magnitude of the injection effect during the control sensory condition (x-axis) for single units (SUs) recorded during scopolamine pressure injection. Red dots show those SUs which were significantly affected by the injected substance, black dots were not. No apparent relationship between the magnitude of attentional modulation of a given cell and the size of the effect of the antagonist injection can be seen. b) As for a, for SUs recorded during injection of agonist acetylcholine.

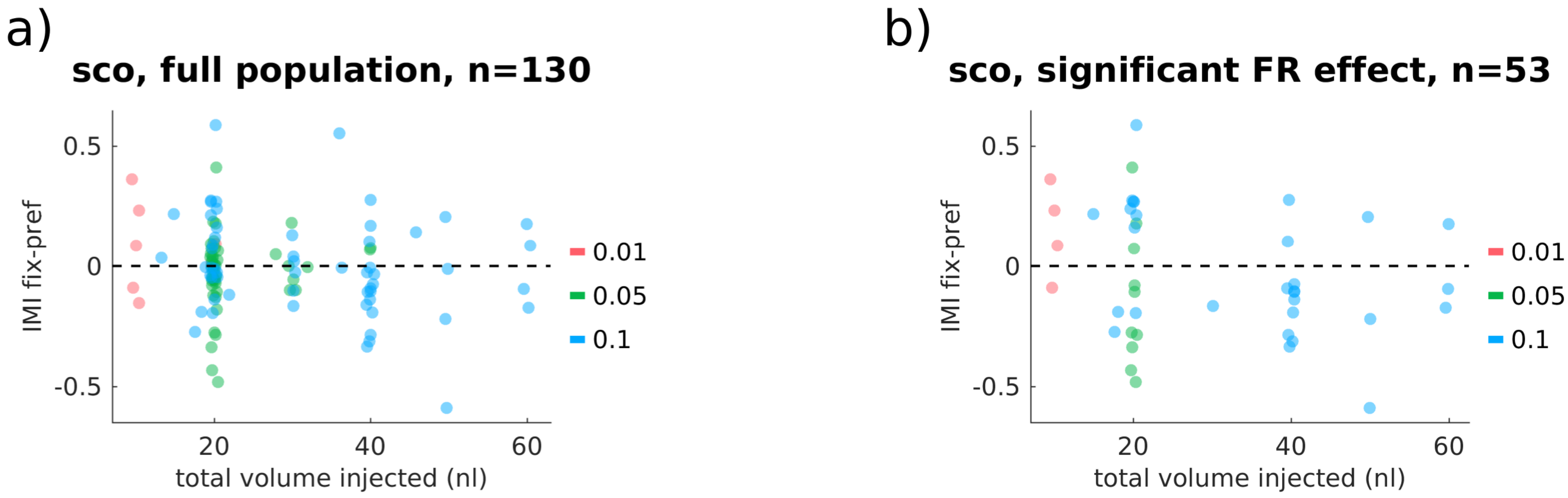

Supplementary Figure 4: No apparent effect of scopolamine volume and concentration. a) Each point depicts a single unit. The color indicates the concentration of scopolamine injected. X-axis plots total volume injected and the y-axis plots the magnitude of the injection effect in the control sensory condition. b) Subset of data plotted in panel a. Only units with significant firing rate modulations by scopolamine injection are plotted.

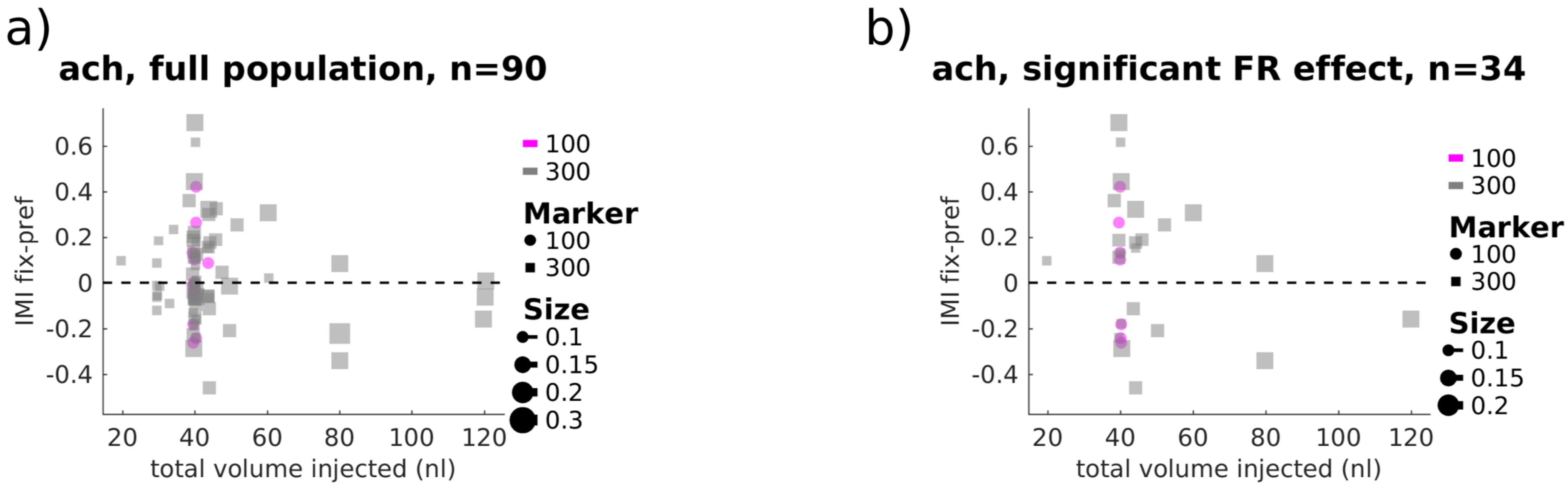

Supplementary Figure 5: No apparent effect of acetylcholine volume and concentration or of inter-tip distance. a) Each point depicts a single unit. The marker and color indicate the inter-tip distance. The size indicates the concentration of acetylcholine injected. X-axis plots total volume injected and the y-axis plots the magnitude of the injection effect in the control sensory condition. b) Subset of data plotted in panel a. Only units with significant firing rate modulations by acetylcholine injection are plotted.

Following pages:

Supplementary Table 1: List of analysed SUs with pharmacological parameters used. List of all cells analyzed with detailed information about the associated recordings.

Supplementary Table 2: AMI and IMI values for analysed SUs. List of all cells analyzed with values of attentional and injection modulation indices for each cell.

Supplementary Table 1

FileName	monkey	hemisphere	substance	concentration	nInjBlocks	totalVolInjected	interTipDistance_um	sigInjEffect	directionOfSigInjEffect
s1-pie-015-01+01_2a	P	L	sco	0.1	3	51	300	1	decrease
s1-pie-022-01+01_3a	P	L	sco	0.1	3	43	300	0	
s1-pie-023-01+02_2a	P	L	sco	0.1	2	30	300	1	increase
s1-pie-023-01+03_2a	P	L	sco	0.1	1	19	300	0	
s1-pie-026-01+01_3a	P	L	sco	0.1	2	31	300	0	
s1-pie-027-01+01_3a	P	L	sco	0.1	1	18	300	1	decrease
s1-pie-030-01+01_2a	P	L	sco	0.1	1	13	300	0	
s1-pie-031-01+01_2a	P	L	sco	0.1	1	18	300	1	decrease
s1-pie-032-01+01_2a	P	L	sco	0.1	2	29	300	1	increase
s1-pie-035-01+01_2a	P	L	sco	0.1	3	41	300	1	increase
s1-pie-037-01+02_2a	P	L	sco	0.01	1	10	300	1	increase
s1-pie-037-01+02_2b	P	L	sco	0.01	1	10	300	1	increase
s1-pie-037-01+02_3a	P	L	sco	0.01	1	10	300	0	
s2-pie-017-02+01_2a	P	R	sco	0.05	2	40	300	0	
s2-pie-018-01+01_1a	P	R	sco	0.05	1	20	300	1	increase
s2-pie-018-01+01_2a	P	R	sco	0.05	1	20	300	1	increase
s2-pie-019-01+01_1a	P	R	sco	0.05	2	40	300	0	
s2-pie-020-01+99_1a	P	R	sco	0.05	2	40	300	0	
s2-pie-023-01+03_1a	P	R	sco	0.05	1	20	300	0	
s2-pie-024-01+02_2b	P	R	sco	0.05	1	20	300	0	
s2-pie-024-02+02_1a	P	R	sco	0.05	1	20	300	0	
s2-pie-035-01+99_1a	P	R	sco	0.05	2	40	300	0	
s2-pie-039-01+02_1a	P	R	sco	0.05	1	20	300	1	decrease
s2-pie-041-02+02_2a	P	R	sco	0.05	1	20	300	0	
s2-pie-044-01+03_2a	P	R	sco	0.05	2	40	300	0	
s2-pie-044-01+03_2b	P	R	sco	0.05	2	40	300	1	decrease
s2-pie-045-01+03_1a	P	R	sco	0.05	1	20	300	0	
s2-pie-045-02+03_1a	P	R	sco	0.05	1	20	300	0	
s2-pie-045-02+03_2a	P	R	sco	0.05	1	20	300	0	
s2-pie-045-02+03_2b	P	R	sco	0.05	1	20	300	0	
s2-pie-048-01+04_1a	P	R	sco	0.05	1	20	300	0	
s2-pie-053-03+02_1a	P	R	sco	0.05	1	32	300	0	
s2-pie-054-01+02_2a	P	R	sco	0.05	1	20	300	1	increase
s2-pie-055-01+02_1a	P	R	sco	0.05	1	20	300	0	
s2-pie-055-01+02_1b	P	R	sco	0.05	1	20	300	0	
s2-pie-055-02+02_1a	P	R	sco	0.05	1	20	300	0	
s2-pie-055-02+02_1b	P	R	sco	0.05	1	20	300	0	

s2-pie-058-01+02_1a	P	R	sco	0.05	1	20	300	0	
s2-pie-059-01+02_1a	P	R	sco	0.05	1	20	300	0	
s2-pie-059-01+02_1b	P	R	sco	0.05	1	20	300	0	
s2-pie-060-01+02_1a	P	R	sco	0.05	1	20	300	0	
s2-pie-061-02+02_1a	P	R	sco	0.05	1	20	300	0	
s2-pie-067-03+01_1a	P	R	sco	0.05	1	30	300	0	
s2-pie-067-03+01_2a	P	R	sco	0.05	1	30	300	0	
s2-pie-075-01+01_1a	P	R	sco	0.05	1	20	300	0	
s3-pie-001-01+02_3a	P	L	sco	0.01	2	20	300	1	increase
s3-pie-001-01+99_2a	P	L	sco	0.01	3	30	300	1	decrease
s3-pie-005-01+01_3a	P	L	sco	0.1	1	20	300	0	
s3-pie-006-01+01_3b	P	L	sco	0.1	1	22	300	0	
s3-pie-009-01+01_3a	P	L	sco	0.01	1	20	300	0	
s3-pie-012-01+01_3a	P	L	sco	0.01	2	40	300	0	
s3-pie-014-01+01_2a	P	L	sco	0.1	1	20	300	0	
s3-pie-014-01+03_2a	P	L	sco	0.1	2	40	300	0	
s3-pie-017-01+01_3a	P	L	sco	0.1	1	40	300	1	decrease
s3-pie-018-01+01_3a	P	L	sco	0.1	3	100	300	1	decrease
s3-pie-018-01+01_3b	P	L	sco	0.1	3	100	300	0	
s3-pie-020-01+01_1a	P	L	sco	0.1	1	40	300	1	decrease
s3-pie-020-01+02_1a	P	L	sco	0.1	1	40	300	1	decrease
s3-pie-021-01+01_1a	P	L	sco	0.05	1	20	300	1	decrease
s3-pie-023-01+01_1a	P	L	sco	0.05	2	30	300	1	increase
s3-pie-023-01+01_3a	P	L	sco	0.05	2	30	300	0	
s3-pie-037-01+01_3a	P	L	sco	0.05	1	40	300	0	
s2-pie-032-02+02_2a	P	R	nacl	0.9	2	40	300	0	
s2-pie-037-01+01_1a	P	R	nacl	0.9	1	24	300	0	
s2-pie-046-01+03_2a	P	R	nacl	0.9	1	20	300	0	
s2-pie-063-03+01_1a	P	R	nacl	0.9	1	20	300	0	
s2-pie-080-01+02_1a	P	R	nacl	0.9	1	20	300	1	decrease
s2-pie-083-02+01_1a	P	R	ach	0.1	1	30	300	0	
s2-pie-085-01+02_2a	P	R	ach	0.1	1	30	300	0	
s2-pie-086-04+01_1a	P	R	ach	0.1	1	33	300	0	
s2-pie-089-02+01_1a	P	R	ach	0.1	2	63	300	1	increase
s2-pie-090-01+02_2a	P	R	ach	0.1	1	30	300	0	
s2-pie-090-01+02_2b	P	R	ach	0.1	1	30	300	0	
s1-osk-125-03+01_1a	O	L	sco	0.1	1	30	100	0	
s1-osk-125-03+01_2a	O	L	sco	0.1	1	30	100	0	

s1-osk-204-02+01_2a	O	L	sco	0.1	1	40	100	0	
s1-osk-205-01+01_2b	O	L	sco	0.1	1	40	100	1	decrease
s1-osk-206-01+01_1a	O	L	sco	0.1	1	40	100	0	
s1-osk-207-02+01_2a	O	L	sco	0.1	1	36	100	0	
s1-osk-207-02+01_2b	O	L	sco	0.1	1	36	100	0	
s1-osk-208-01+01_1a	O	L	sco	0.1	1	50	100	0	
s1-osk-208-01+01_2a	O	L	sco	0.1	1	50	100	1	decrease
s1-osk-209-01+01_1a	O	L	sco	0.1	1	40	100	1	increase
s1-osk-210-01+01_1a	O	L	sco	0.1	1	46	100	0	
s1-osk-211-01+01_1a	O	L	sco	0.1	1	50	100	1	decrease
s1-osk-211-01+01_2a	O	L	sco	0.1	1	50	100	1	increase
s1-osk-212-01+01_1a	O	L	sco	0.1	1	40	100	1	decrease
s1-osk-213-01+01_1a	O	L	sco	0.1	1	40	100	0	
s1-osk-214-01+01_1a	O	L	sco	0.1	1	40	100	1	decrease
s1-osk-215-01+01_2a	O	L	sco	0.1	1	40	100	1	decrease
s2-osk-010-02+01_2a	O	R	sco	0.05	1	20	300	1	decrease
s2-osk-013-03+02_2a	O	R	sco	0.05	2	40	300	0	
s2-osk-014-03+01_2a	O	R	sco	0.05	1	30	300	0	
s2-osk-015-01+01_2a	O	R	sco	0.05	1	30	300	0	
s2-osk-016-03+01_1a	O	R	sco	0.05	1	28	300	0	
s2-osk-018-03+01_1a	O	R	sco	0.05	1	30	300	0	
s2-osk-019-01+01_2a	O	R	sco	0.05	1	20	300	0	
s2-osk-021-02+01_2a	O	R	sco	0.05	1	20	300	0	
s2-osk-022-01+01_1a	O	R	sco	0.05	1	20	300	1	decrease
s2-osk-022-01+01_2a	O	R	sco	0.05	1	20	300	1	decrease
s2-osk-022-01+01_2b	O	R	sco	0.05	1	20	300	0	
s2-osk-023-01+01_1a	O	R	sco	0.05	1	40	300	0	
s2-osk-025-01+01_2a	O	R	sco	0.05	2	40	300	0	
s2-osk-026-01+01_2a	O	R	sco	0.05	3	60	300	1	decrease
s2-osk-029-02+01_1a	O	R	sco	0.1	1	20	300	0	
s2-osk-029-02+01_1b	O	R	sco	0.1	1	20	300	1	decrease
s2-osk-029-02+01_2a	O	R	sco	0.1	1	20	300	1	increase
s2-osk-030-01+01_1a	O	R	sco	0.1	2	50	300	0	
s2-osk-031-01+01_1a	O	R	sco	0.1	1	20	300	1	increase
s2-osk-032-01+01_1a	O	R	sco	0.1	1	20	300	0	
s2-osk-034-01+01_1a	O	R	sco	0.1	1	20	300	0	
s2-osk-036-01+01_1a	O	R	sco	0.1	1	20	300	0	
s2-osk-038-01+01_1a	O	R	sco	0.1	1	20	300	1	increase

s2-osk-038-01+01_1b	O	R	sco	0.1	1	20	300	1	increase
s2-osk-038-02+01_1a	O	R	sco	0.1	1	30	300	0	
s2-osk-039-01+01_2a	O	R	sco	0.1	1	30	300	0	
s2-osk-040-01+01_2b	O	R	sco	0.1	1	20	300	1	increase
s2-osk-040-01+99_2a	O	R	sco	0.1	2	40	300	0	
s2-osk-041-02+01_1a	O	R	sco	0.1	1	20	300	0	
s2-osk-041-02+01_1b	O	R	sco	0.1	1	20	300	1	increase
s2-osk-042-02+01_1a	O	R	sco	0.1	2	50	300	0	
s2-osk-042-02+01_2a	O	R	sco	0.1	2	50	300	0	
s2-osk-048-01+01_1a	O	R	sco	0.1	2	50	300	0	
s2-osk-049-01+01_1a	O	R	sco	0.1	2	40	300	0	
s2-osk-049-01+01_2b	O	R	sco	0.1	2	40	300	1	increase
s2-osk-051-01+01_2a	O	R	sco	0.1	3	70	300	1	increase
s2-osk-052-01+01_2a	O	R	sco	0.1	2	50	300	1	increase
s2-osk-053-01+01_1a	O	R	sco	0.1	1	30	300	1	decrease
s2-osk-054-02+01_2a	O	R	sco	0.1	1	30	300	0	
s2-osk-055-01+01_1a	O	R	sco	0.1	2	80	300	0	
s2-osk-060-02+01_2a	O	R	sco	0.1	1	60	300	1	decrease
s2-osk-062-01+01_2a	O	R	sco	0.1	1	40	300	1	decrease
s2-osk-062-03+01_1a	O	R	sco	0.1	1	40	300	1	decrease
s2-osk-070-01+01_1a	O	R	sco	0.1	1	60	300	1	increase
s2-osk-071-01+01_2a	O	R	sco	0.1	1	40	300	0	
s2-osk-071-02+01_1a	O	R	sco	0.1	1	40	300	1	increase
s2-osk-072-01+01_1a	O	R	sco	0.1	1	60	300	1	decrease
s2-osk-073-01+01_2a	O	R	sco	0.1	2	100	300	1	decrease
s2-osk-073-01+01_2b	O	R	sco	0.1	2	100	300	1	decrease
s2-osk-075-01+01_2a	O	R	sco	0.1	2	100	300	1	increase
s2-osk-076-01+01_2a	O	R	sco	0.1	1	60	300	0	
s1-osk-202-01+01_2a	O	L	nacl	0.9	1	40	100	0	
s2-osk-006-02+01_1a	O	R	nacl	0.9	2	40	300	0	
s2-osk-006-02+01_1b	O	R	nacl	0.9	2	40	300	1	increase
s2-osk-007-01+02_2a	O	R	nacl	0.9	1	20	300	0	
s2-osk-008-01+01_2a	O	R	nacl	0.9	1	20	300	0	
s2-osk-011-02+01_2a	O	R	nacl	0.9	1	20	300	0	
s2-osk-044-01+01_2a	O	R	nacl	0.9	1	20	300	0	
s2-osk-044-02+01_2a	O	R	nacl	0.9	1	20	300	1	increase
s2-osk-044-02+01_2b	O	R	nacl	0.9	1	20	300	0	
s2-osk-044-03+01_2a	O	R	nacl	0.9	1	30	300	0	

s2-osk-045-01+02_1a	O	R	nacl	0.9	2	40	300	0	
s2-osk-045-01+02_2a	O	R	nacl	0.9	2	40	300	1	decrease
s2-osk-046-01+01_2a	O	R	nacl	0.9	2	50	300	0	
s2-osk-066-01+01_1b	O	R	nacl	0.9	1	32	300	0	
s2-osk-067-01+01_1a	O	R	nacl	0.9	1	40	300	0	
s2-osk-067-02+01_1a	O	R	nacl	0.9	1	40	300	0	
s2-osk-068-01+01_1a	O	R	nacl	0.9	1	40	300	0	
s2-osk-069-01+01_1a	O	R	nacl	0.9	1	40	300	0	
s2-osk-074-01+01_1a	O	R	nacl	0.9	1	60	300	1	decrease
s2-osk-074-01+01_1b	O	R	nacl	0.9	1	60	300	1	decrease
s2-osk-074-01+01_2a	O	R	nacl	0.9	1	60	300	0	
s2-osk-074-02+01_1a	O	R	nacl	0.9	1	60	300	0	
s2-osk-087-01+01_2a	O	R	nacl	0.9	1	30	300	0	
s1-osk-090-01+01_2a	O	L	ach	0.1	1	40	300	0	
s1-osk-090-01+01_2b	O	L	ach	0.1	1	40	300	0	
s1-osk-091-01+01_2a	O	L	ach	0.1	1	30	300	0	
s1-osk-092-01+01_2a	O	L	ach	0.1	1	40	300	0	
s1-osk-094-01+01_1a	O	L	ach	0.1	1	40	300	0	
s1-osk-095-01+01_2a	O	L	ach	0.1	1	40	300	0	
s1-osk-095-01+01_2b	O	L	ach	0.1	1	40	300	0	
s1-osk-098-01+02_2b	O	L	ach	0.2	1	40	300	1	increase
s1-osk-100-02+01_1a	O	L	ach	0.2	1	40	300	0	
s1-osk-106-01+01_1a	O	L	ach	0.15	2	80	300	0	
s1-osk-107-02+01_1a	O	L	ach	0.15	1	40	300	0	
s1-osk-109-01+01_1a	O	L	ach	0.15	1	40	300	0	
s1-osk-110-01+01_1a	O	L	ach	0.15	1	40	300	0	
s1-osk-115-01+01_1a	O	L	ach	0.15	1	40	300	0	
s1-osk-115-01+01_1b	O	L	ach	0.15	1	40	300	0	
s1-osk-116-01+01_1a	O	L	ach	0.15	1	40	300	0	
s1-osk-117-01+01_1a	O	L	ach	0.15	3	140	300	0	
s1-osk-130-01+01_1a	O	L	ach	0.2	1	60	300	1	increase
s1-osk-132-01+01_2a	O	L	ach	0.2	1	44	300	1	increase
s1-osk-134-02+01_1b	O	L	ach	0.2	1	40	300	1	increase
s1-osk-134-03+01_1a	O	L	ach	0.2	1	50	300	0	
s1-osk-136-01+01_2a	O	L	ach	0.2	2	380	300	1	increase
s1-osk-143-01+01_2b	O	L	ach	0.3	1	80	300	0	
s1-osk-145-01+01_1a	O	L	ach	0.2	1	80	300	1	increase
s1-osk-145-02+01_1a	O	L	ach	0.2	1	80	300	1	decrease

s1-osk-147-01+01_1a	O	L	ach	0.2	1	120	300	0	
s1-osk-147-01+01_2a	O	L	ach	0.2	1	120	300	1	decrease
s1-osk-147-01+01_2b	O	L	ach	0.2	1	120	300	0	
s1-osk-150-01+01_1a	O	L	ach	0.15	1	40	300	0	
s1-osk-150-01+01_1b	O	L	ach	0.15	1	40	300	1	increase
s1-osk-150-01+01_1c	O	L	ach	0.15	1	40	300	0	
s1-osk-155-01+01_1a	O	L	ach	0.15	1	40	300	0	
s1-osk-155-01+01_1b	O	L	ach	0.15	1	40	300	1	increase
s1-osk-155-02+01_1a	O	L	ach	0.15	1	44	300	1	decrease
s1-osk-155-02+01_1b	O	L	ach	0.15	1	44	300	0	
s1-osk-157-01+01_2a	O	L	ach	0.15	1	48	300	0	
s1-osk-159-01+01_1a	O	L	ach	0.15	1	44	300	1	increase
s1-osk-159-01+01_1b	O	L	ach	0.15	1	44	300	1	decrease
s1-osk-160-01+01_1a	O	L	ach	0.15	1	44	300	0	
s1-osk-160-01+01_1b	O	L	ach	0.15	1	44	300	0	
s1-osk-160-02+01_1a	O	L	ach	0.15	1	52	300	1	increase
s1-osk-162-01+01_2a	O	L	ach	0.15	1	50	300	1	decrease
s1-osk-164-01+01_2b	O	L	ach	0.15	1	44	300	0	
s1-osk-165-01+01_2a	O	L	ach	0.15	1	38	300	1	increase
s1-osk-166-01+01_1a	O	L	ach	0.15	1	46	300	0	
s1-osk-166-01+01_2a	O	L	ach	0.15	1	46	300	1	increase
s1-osk-167-01+01_1a	O	L	ach	0.1	2	56	300	1	decrease
s1-osk-167-01+01_2a	O	L	ach	0.1	2	56	300	1	decrease
s1-osk-168-01+01_2a	O	L	ach	0.1	1	60	300	0	
s1-osk-169-01+01_1a	O	L	ach	0.1	1	30	300	0	
s1-osk-169-01+01_2a	O	L	ach	0.1	1	30	300	0	
s1-osk-177-01+01_2a	O	L	ach	0.1	1	34	300	0	
s1-osk-180-01+01_1a	O	L	ach	0.1	1	40	300	0	
s1-osk-180-01+01_2a	O	L	ach	0.1	1	40	300	0	
s1-osk-182-01+01_1a	O	L	ach	0.1	1	44	300	1	increase
s1-osk-182-01+01_2a	O	L	ach	0.1	1	44	300	0	
s1-osk-184-01+01_2a	O	L	ach	0.1	1	40	300	1	decrease
s1-osk-184-01+01_2b	O	L	ach	0.1	1	40	300	0	
s1-osk-185-01+01_2a	O	L	ach	0.1	1	40	300	1	increase
s1-osk-187-01+01_1a	O	L	ach	0.1	1	40	300	0	
s1-osk-187-01+01_1b	O	L	ach	0.1	1	40	300	0	
s1-osk-188-01+01_1a	O	L	ach	0.1	1	40	300	0	
s1-osk-188-01+01_1b	O	L	ach	0.1	1	40	300	0	

s1-osk-189-01+01_1a	O	L	ach	0.1	1	40	300	1	decrease
s1-osk-189-01+01_1b	O	L	ach	0.1	1	40	300	1	increase
s1-osk-191-01+01_1d	O	L	ach	0.2	1	40	300	0	
s1-osk-191-01+01_2a	O	L	ach	0.2	1	40	300	1	decrease
s1-osk-192-01+01_1a	O	L	ach	0.1	1	40	100	1	increase
s1-osk-193-01+01_1c	O	L	ach	0.1	1	40	100	1	decrease
s1-osk-194-01+01_1a	O	L	ach	0.1	1	40	100	1	increase
s1-osk-195-01+01_1a	O	L	ach	0.1	1	40	100	1	increase
s1-osk-195-01+01_1b	O	L	ach	0.1	1	40	100	0	
s1-osk-196-01+01_1a	O	L	ach	0.1	1	40	100	1	decrease
s1-osk-198-01+01_2a	O	L	ach	0.1	1	40	100	1	decrease
s1-osk-199-01+01_1a	O	L	ach	0.1	1	40	100	0	
s1-osk-199-01+01_1c	O	L	ach	0.1	1	40	100	0	
s1-osk-199-02+01_1a	O	L	ach	0.1	1	44	100	0	
s1-osk-200-01+01_1a	O	L	ach	0.1	1	40	100	1	increase
s1-osk-200-01+02_1a	O	L	ach	0.1	1	40	100	0	
s2-osk-082-01+01_2a	O	R	ach	0.1	1	20	300	1	increase
s2-osk-083-01+01_2a	O	R	ach	0.1	1	40	300	0	
s2-osk-084-01+01_1a	O	R	ach	0.1	2	80	300	0	
s2-osk-086-01+01_1a	O	R	ach	0.1	2	70	300	0	
s2-osk-088-01+01_2a	O	R	ach	0.1	2	60	300	0	

Supplementary Table 2

FileName	substance	AMI_control	AMI_injection	IMI_attFix	IMI_attIn	IMI_attOut
s1-pie-015-01+01_2a	sco	-0.0535580895	0.0354876822	-0.1086334232	-0.1060722489	-0.1931284042
s1-pie-022-01+01_3a	sco	0.1684081986	0.1729437949	0.1334329077	0.2379867988	0.2335748267
s1-pie-023-01+02_2a	sco	-0.0499473646	-0.009650813	0.2185506481	0.1341110185	0.0943049223
s1-pie-023-01+03_2a	sco	0.0691857939	0.0084008352	-0.003770219	-0.04499995	0.0158637763
s1-pie-026-01+01_3a	sco	-0.0561741515	0.1006524708	0.1177187484	0.2263121175	0.0729414851
s1-pie-027-01+01_3a	sco	-0.1005017253	-0.018357868	-0.1906469688	-0.0970207441	-0.1778960451
s1-pie-030-01+01_2a	sco	0.1352394995	-0.031556365	0.0333341758	-0.0807822252	0.0864649247
s1-pie-031-01+01_2a	sco	0.132882906	0.411990571	-0.2732734137	-0.0835159544	-0.3696726498
s1-pie-032-01+01_2a	sco	0.1606691971	0.104427086	0.1629709371	0.085140681	0.1416526597
s1-pie-035-01+01_2a	sco	0.2046446459	0.0989244229	0.0812719204	0.0669160185	0.1735674493
s1-pie-037-01+02_2a	sco	0.1276151147	0.0005161907	0.3606726632	0.0849981866	0.2098384125
s1-pie-037-01+02_2b	sco	0.1000222711	0.0602566242	0.2318032397	0.2043897413	0.242414294
s1-pie-037-01+02_3a	sco	-0.0189372934	0.0956490149	-0.1543365484	-0.101168773	-0.2130822014
s2-pie-017-02+01_2a	sco	-0.0591230909	0.0242385607	-0.0610298914	-0.0095129593	-0.0926819266
s2-pie-018-01+01_1a	sco	0.2496468493	0.1617679447	0.4109990174	0.1734660337	0.2608987544
s2-pie-018-01+01_2a	sco	0.1354624743	0.2605633791	0.1784745807	0.2199598376	0.0929325476
s2-pie-019-01+01_1a	sco	0.2218439392	0.1385286226	0.0423725161	0.1062993418	0.1905154925
s2-pie-020-01+99_1a	sco	0.0916579292	0.128989053	-0.0606797504	-0.0281678847	-0.0658755506
s2-pie-023-01+03_1a	sco	-0.1338691196	0.2032100802	-0.1228847993	0.2229351043	-0.1135220839
s2-pie-024-01+02_2b	sco	0.069584343	-0.165621834	0.093179255	0.0238502738	0.2549626803
s2-pie-024-02+02_1a	sco	0.2216268666	0.1414651673	0.0360650853	-0.0342559247	0.0486382748
s2-pie-035-01+99_1a	sco	0.024412149	0.0264167339	-0.0596157196	0.0186746824	0.0166694283
s2-pie-039-01+02_1a	sco	0.1031889734	0.1822044701	-0.079937199	0.0130317484	-0.0675687338
s2-pie-041-02+02_2a	sco	0.2396362526	0.1682666535	0.0323031119	0.0640754306	0.1377871874
s2-pie-044-01+03_2a	sco	0.0474230171	0.1519093948	-0.0230888755	0.0260845426	-0.0793779299
s2-pie-044-01+03_2b	sco	0.1488447522	0.0680394575	-0.3359699214	-0.2114597651	-0.1321081938
s2-pie-045-01+03_1a	sco	0.064606136	-0.082714219	0.1854966049	0.1108094296	0.2532347582
s2-pie-045-02+03_1a	sco	-0.0107628267	0.1135970578	-0.0454167641	0.072268349	-0.0524101251
s2-pie-045-02+03_2a	sco	0.1289653164	0.4797889905	0.0185880416	0.0707261286	-0.3114751292
s2-pie-045-02+03_2b	sco	0.147513426	0.0904384365	-0.0713458228	-0.1168023375	-0.0593566729
s2-pie-048-01+04_1a	sco	0.1087153409	0.128728729	-0.0169709283	0.1114611793	0.0913704473
s2-pie-053-03+02_1a	sco	0.0053928438	-0.228270598	-0.0058133218	-0.2559050764	-0.0239598612
s2-pie-054-01+02_2a	sco	0.0835706584	0.004402266	0.0736791485	-0.0255889777	0.0537174143
s2-pie-055-01+02_1a	sco	0.0693711963	-0.053786334	0.0011145707	-0.071622962	0.0515295965
s2-pie-055-01+02_1b	sco	0.018865664	0.2776144926	0.0757438357	0.1228099766	-0.1418318647
s2-pie-055-02+02_1a	sco	0.0080269057	-0.118798682	-0.0521561807	-0.0729931822	0.0542129753

s2-pie-055-02+02_1b	sco	0.0247116426	-0.039498411	0.005895051	-0.0183206688	0.0458806921
s2-pie-058-01+02_1a	sco	-0.0126372885	0.0720914902	0.0248274482	0.11009374	0.0256814236
s2-pie-059-01+02_1a	sco	0.1560631492	0.149883057	0.0036870258	-0.0982243782	-0.0919534197
s2-pie-059-01+02_1b	sco	0.057231626	0.1089427683	-0.1286942841	0.08819621	0.036327346
s2-pie-060-01+02_1a	sco	-0.0961176153	-0.033777467	0.0628823048	0.0057183089	-0.0568452233
s2-pie-061-02+02_1a	sco	0.0826487949	-0.119018998	-0.0398083995	-0.2348195305	-0.0368439444
s2-pie-067-03+01_1a	sco	-0.1008923798	-0.044775386	-0.0569464169	0.0813083032	0.0250514741
s2-pie-067-03+01_2a	sco	-0.1454186288	-0.070000444	-0.1003021788	0.0022522605	-0.0739542206
s2-pie-075-01+01_1a	sco	0.102155949	0.164761913	0.0518172325	0.1213920452	0.0581639003
s3-pie-001-01+02_3a	sco	0.0558461579	0.0861090317	0.0844617296	-0.0296453335	-0.0600003508
s3-pie-001-01+99_2a	sco	0.103534238	0.1828660151	-0.0903613489	0.009001855	-0.0719132345
s3-pie-005-01+01_3a	sco	-0.0223381173	-0.119541604	-0.0263757033	-0.0571511013	0.0405384514
s3-pie-006-01+01_3b	sco	0.1488361614	0.1579443683	-0.1207771501	-0.0567480709	-0.0660405902
s3-pie-009-01+01_3a	sco	0.3291881623	0.2838203903	0.0140216512	-0.1143477137	-0.0646744664
s3-pie-012-01+01_3a	sco	0.0787003091	-0.084405223	0.0940543961	-0.0569896906	0.1060185018
s3-pie-014-01+01_2a	sco	0.1914999215	0.214799178	0.0745460532	0.0253425827	0.0010444635
s3-pie-014-01+03_2a	sco	0.0091859043	0.0425318503	-0.0113212677	0.01432863	-0.0190394498
s3-pie-017-01+01_3a	sco	0.1997246157	0.3350543798	-0.2847044865	-0.2738563046	-0.4028893343
s3-pie-018-01+01_3a	sco	-0.0536633898	-0.141213575	-0.4821966246	-0.480895877	-0.410074162
s3-pie-018-01+01_3b	sco	0.6463003282	0.6247583767	0.1157315201	0.1108507578	0.1463953936
s3-pie-020-01+01_1a	sco	0.0958865489	0.2137908434	-0.0749070119	-0.0621488451	-0.1811654292
s3-pie-020-01+02_1a	sco	0.2075340296	0.4464100097	-0.1933280226	-0.2636841966	-0.4927446315
s3-pie-021-01+01_1a	sco	0.5401214125	0.6659849088	-0.4316288168	-0.1359040244	-0.3238264333
s3-pie-023-01+01_1a	sco	0.1730205352	0.0973204802	0.3599426272	0.3097961967	0.3777814422
s3-pie-023-01+01_3a	sco	0.1663966396	0.1577383647	0.0342387739	0.0679828744	0.0768280887
s3-pie-037-01+01_3a	sco	0.1410575047	0.0075185489	0.0746677109	-0.0434353762	0.090772422
s2-pie-032-02+02_2a	nacl	0.275736928	0.2086985825	-0.1059655827	-0.017241014	0.0539568403
s2-pie-037-01+01_1a	nacl	-0.2886401817	0.0317931518	-0.2433964859	0.2873786692	-0.0331673199
s2-pie-046-01+03_2a	nacl	-0.0446717556	0.0130029433	0.0263583361	-0.0335384673	-0.0910037561
s2-pie-063-03+01_1a	nacl	0.0313894971	0.181764415	-0.0382616005	0.0268878717	-0.1248576615
s2-pie-080-01+02_1a	nacl	0.118084729	0.0167586396	-0.1606386976	-0.0293421274	0.0724005604
s2-pie-083-02+01_1a	ach	0.1300305874	0.2937489234	-0.120253322	0.1207888292	-0.0504689701
s2-pie-085-01+02_2a	ach	0.0396646716	0.2730685045	-0.0621885815	0.0322394666	-0.2052817059
s2-pie-086-04+01_1a	ach	0.0100410413	0.0235325675	-0.0906480982	0.0318500391	0.0183632168
s2-pie-089-02+01_1a	ach	0.0966340422	0.060668538	0.2961922074	0.2115548916	0.2458508608
s2-pie-090-01+02_2a	ach	0.0865946054	0.1366521658	-0.0143169979	0.1264042656	0.076235419
s2-pie-090-01+02_2b	ach	-0.0133903522	-0.012471768	-0.0128753132	-0.0604088404	-0.0613241741

s1-osk-125-03+01_1a	sco	-0.0370274422	0.0781399311	0.0201979595	0.0914748859	-0.0236082259
s1-osk-125-03+01_2a	sco	0.0334230305	0.0516974248	-0.0268345998	-0.0106638326	-0.0289642034
s1-osk-204-02+01_2a	sco	0.2284723612	-0.119928067	-0.0266412986	-0.1755599876	0.1739018356
s1-osk-205-01+01_2b	sco	0.06482903	0.1125838046	-0.3136747753	-0.4002879422	-0.4399225874
s1-osk-206-01+01_1a	sco	0.0054937494	0.0406172917	-0.1601130138	-0.10821416	-0.1428026468
s1-osk-207-02+01_2a	sco	0.016453871	-0.023536538	-0.0071987684	-0.0374498792	0.0025288344
s1-osk-207-02+01_2b	sco	-0.2675976826	-0.193802243	0.5533445538	0.8072661947	0.7783381116
s1-osk-208-01+01_1a	sco	0.0342612234	0.0318270779	-0.0117354326	0.0616148866	0.0640420738
s1-osk-208-01+01_2a	sco	0.185577271	0.3576704333	-0.5894664432	-0.1415770995	-0.3176164102
s1-osk-209-01+01_1a	sco	0.2022327082	0.0338481985	0.2773392685	0.2373025315	0.3911118247
s1-osk-210-01+01_1a	sco	0.0326635667	-0.106268146	0.1421023391	-0.1654651099	-0.0276473431
s1-osk-211-01+01_1a	sco	0.1190445993	0.1598736291	-0.2206897385	-0.1984117436	-0.2380669241
s1-osk-211-01+01_2a	sco	-0.0564654158	0.1545429084	0.2042289234	0.2403609402	0.0328286186
s1-osk-212-01+01_1a	sco	0.1037600969	0.0147188703	-0.3349725666	-0.4429967287	-0.3683719543
s1-osk-213-01+01_1a	sco	0.1919575354	0.1369335826	-0.0061910111	-0.0677711303	-0.0113051006
s1-osk-214-01+01_1a	sco	0.0909248457	0.0757559967	-0.1068357882	-0.1255061286	-0.1104437897
s1-osk-215-01+01_2a	sco	0.3032345634	0.0324809407	-0.1395671398	-0.2977538723	-0.0264614663
s2-osk-010-02+01_2a	sco	0.075377177	-0.092647203	-0.2866225486	-0.4025632446	-0.2526767615
s2-osk-013-03+02_2a	sco	0.0792575715	-0.001745245	0.0036772347	-0.0618541851	0.0192337832
s2-osk-014-03+01_2a	sco	0.0616022149	0.1713205709	0.1803425042	0.1143746724	0.0035308088
s2-osk-015-01+01_2a	sco	0.0933582509	0.0720750359	-0.0997601985	-0.0681676035	-0.0468085789
s2-osk-016-03+01_1a	sco	0.0454214464	0.0795492573	0.0486638694	0.0487617393	0.0145344441
s2-osk-018-03+01_1a	sco	0.0615727334	0.0891140229	0.0012959108	-0.0573437201	-0.0849021349
s2-osk-019-01+01_2a	sco	0.0491601612	0.0582235559	-0.0542074296	0.004365278	-0.0047243206
s2-osk-021-02+01_2a	sco	0.0683044568	0.2748621331	-0.0459680273	-0.0151323625	-0.2249257124
s2-osk-022-01+01_1a	sco	-0.198389419	0.1641791885	-0.4816839014	-0.2297254357	-0.537500317
s2-osk-022-01+01_2a	sco	-0.0165870494	-0.01009371	-0.2754221934	-0.1556933938	-0.1620239923
s2-osk-022-01+01_2b	sco	0.020856063	-0.052800953	-0.181785562	0.2282142653	0.2968065519
s2-osk-023-01+01_1a	sco	0.0181740847	-0.151798494	0.0690691194	-0.1988791187	-0.0303989487
s2-osk-025-01+01_2a	sco	-0.0036356569	0.1639302779	0.1041878452	-0.0284923493	-0.195027899
s2-osk-026-01+01_2a	sco	0.0289948691	0.0387378097	-0.1074664819	0.0235154672	0.0137647282
s2-osk-029-02+01_1a	sco	-0.1759479981	0.1620582441	-0.0472868296	0.2519923192	-0.0835634605
s2-osk-029-02+01_1b	sco	0.0455443316	-0.021508295	-0.1953976932	0.0717521875	0.1380755401
s2-osk-029-02+01_2a	sco	0.1730860454	0.215049821	0.2130757387	0.0785625004	0.0350965348
s2-osk-030-01+01_1a	sco	0.1033668609	0.1056939861	0.0434661447	0.0584024959	0.0560573683
s2-osk-031-01+01_1a	sco	0.1075144227	0.1863072273	0.2404162494	0.0391011851	-0.0414324137
s2-osk-032-01+01_1a	sco	-0.0149557408	-0.159315638	0.0872193647	-0.2549289315	-0.1144460979

s2-osk-034-01+01_1a	sco	0.0120392572	0.081454124	-0.0379990963	0.0068217986	-0.0626909219
s2-osk-036-01+01_1a	sco	-0.1068509611	-0.256225117	-0.0493418714	-0.1936375495	-0.041286533
s2-osk-038-01+01_1a	sco	-0.0400587484	0.0419256216	0.2681207142	0.2086381879	0.1289940313
s2-osk-038-01+01_1b	sco	0.1098122481	0.1091722414	0.2688508676	0.2213283736	0.2219443259
s2-osk-038-02+01_1a	sco	-0.0342778505	0.0918929131	0.1287703847	0.036990774	-0.0891988108
s2-osk-039-01+01_2a	sco	0.2253163869	0.2283011204	-0.101360867	0.0705025126	0.067370864
s2-osk-040-01+01_2b	sco	0.2633345118	-0.164476982	0.5872331336	0.3938409461	0.6921184808
s2-osk-040-01+99_2a	sco	-0.0822778188	0.0306561872	-0.1388623531	-0.0453935853	-0.1572393954
s2-osk-041-02+01_1a	sco	-0.0982629373	-0.217394618	0.1195052443	-0.2681884039	-0.1513990481
s2-osk-041-02+01_1b	sco	0.0331190108	0.3051040883	0.1618740512	0.0690559752	-0.2096840252
s2-osk-042-02+01_1a	sco	0.0595081027	0.1258781683	0.0405957072	0.0109891072	-0.0559229687
s2-osk-042-02+01_2a	sco	-0.023621809	0.071792917	0.0119612224	0.0100264499	-0.085308212
s2-osk-048-01+01_1a	sco	0.0303525801	-0.023622559	-0.0273680273	-0.0113370897	0.042625441
s2-osk-049-01+01_1a	sco	0.1322065735	0.0301288234	-0.0068864621	-0.0217306967	0.0809355293
s2-osk-049-01+01_2b	sco	-0.1066732545	0.1442394544	0.2737100722	0.466122429	0.2475224532
s2-osk-051-01+01_2a	sco	0.1248383093	0.0957174246	0.0919449091	0.0175458799	0.0469946419
s2-osk-052-01+01_2a	sco	0.0136148539	0.0063453899	0.0807914205	0.0870784871	0.0942888879
s2-osk-053-01+01_1a	sco	0.138358665	0.13039579	-0.1652967225	-0.0627757153	-0.0546943822
s2-osk-054-02+01_2a	sco	-0.0351264252	0.0452238512	0.0387706543	0.1150046975	0.0351057449
s2-osk-055-01+01_1a	sco	0.3226965306	-0.018164732	0.1684208329	-0.1230764807	0.2251905412
s2-osk-060-02+01_2a	sco	0.1135081837	-0.439915988	-0.1745677694	-0.7664816496	-0.4016511292
s2-osk-062-01+01_2a	sco	0.1321904991	-0.029127985	-0.1038396343	-0.2375413594	-0.0798913178
s2-osk-062-03+01_1a	sco	0.0806662247	0.0053777478	-0.091966614	-0.0859970271	-0.0107454784
s2-osk-070-01+01_1a	sco	0.2488165493	0.1905675511	0.1768702035	0.180570419	0.2390790386
s2-osk-071-01+01_2a	sco	0.0498270684	0.1336991708	-0.034371633	0.0341931808	-0.0503868825
s2-osk-071-02+01_1a	sco	-0.1709051074	0.0009974995	0.1027041132	0.0412431651	-0.1315627367
s2-osk-072-01+01_1a	sco	0.0146115857	0.0379942621	-0.0944142076	-0.0758185906	-0.099038578
s2-osk-073-01+01_2a	sco	0.1718233307	0.1276917694	-0.3203660275	-0.3013747765	-0.2597859272
s2-osk-073-01+01_2b	sco	-0.0527759943	0.1033465372	-0.1305953865	-0.0671519788	-0.2201322698
s2-osk-075-01+01_2a	sco	0.1593267997	-0.015602188	0.0738346731	-0.0008498375	0.1736711355
s2-osk-076-01+01_2a	sco	-0.0031870377	0.0026272223	0.0847076358	0.0367260387	0.0309184295
s1-osk-202-01+01_2a	nacl	-0.0083520001	-0.066674758	-0.0381751838	-0.0422883814	0.0161066194
s2-osk-006-02+01_1a	nacl	0.0728252951	0.1911808313	-0.1150679604	0.0288059057	-0.0915372253
s2-osk-006-02+01_1b	nacl	-0.101186927	-0.384282057	0.1217412398	-0.0852829459	0.2146576924
s2-osk-007-01+02_2a	nacl	-0.0864650344	-0.041678442	-0.104721591	0.0047287003	-0.0402284246
s2-osk-008-01+01_2a	nacl	0.0204161639	-0.150009994	-0.0074507402	-0.0347432758	0.1359651374
s2-osk-011-02+01_2a	nacl	0.2451809436	0.2082803642	0.1214962463	0.0393902081	0.0781568572

s2-osk-044-01+01_2a	nacl	-0.0574538829	-0.07442983	-0.0932299502	-0.0060957934	0.0109541983
s2-osk-044-02+01_2a	nacl	0.0526484358	0.1230617542	0.0660356791	0.0489680225	-0.0219807634
s2-osk-044-02+01_2b	nacl	0.1390773123	0.1231485901	0.0358877684	0.0469879042	0.0631461086
s2-osk-044-03+01_2a	nacl	0.1560158198	0.0129789167	0.0321037226	-0.0530244156	0.0909942545
s2-osk-045-01+02_1a	nacl	0.1351249782	0.1488386449	0.1221415877	0.0036125161	-0.0103831432
s2-osk-045-01+02_2a	nacl	0.1563908529	0.0897934974	-0.3529952889	-0.4001993348	-0.3418954986
s2-osk-046-01+01_2a	nacl	0.0486871325	0.0358668082	-0.0489697038	-0.1059493917	-0.0932335019
s2-osk-066-01+01_1b	nacl	0.2842487891	0.128925535	0.0355912405	-0.0492913254	0.1128373387
s2-osk-067-01+01_1a	nacl	0.1040603555	0.1167698244	0.0049279894	0.1697868052	0.1572645379
s2-osk-067-02+01_1a	nacl	0.0676050881	0.0391604241	-0.0377914028	-0.1026796654	-0.0743773053
s2-osk-068-01+01_1a	nacl	0.041206875	0.0898598388	-0.0940777893	0.0326166654	-0.016242994
s2-osk-069-01+01_1a	nacl	-0.2707107441	-0.015088177	-0.1110984372	0.1699240547	-0.0907028588
s2-osk-074-01+01_1a	nacl	0.0377862258	0.4177375802	-0.8096061614	-0.835234139	-0.923505802
s2-osk-074-01+01_1b	nacl	0.1922722129	0.0103465417	-0.5119070603	-0.6346992269	-0.5116024239
s2-osk-074-01+01_2a	nacl	0.1408290539	0.0568666825	0.0320146839	-0.0186430144	0.0661015012
s2-osk-074-02+01_1a	nacl	0.0178460913	-0.09483382	0.1015450413	0.0723688233	0.1833656245
s2-osk-087-01+01_2a	nacl	0.1962014408	0.1843687497	0.0181929659	0.0579234472	0.0701503467
s1-osk-090-01+01_2a	ach	-0.1244382502	-0.222646524	0.1075533402	0.0067956192	0.1077284143
s1-osk-090-01+01_2b	ach	0.2392497064	0.0121062507	0.1825292522	-0.0432484673	0.1863911537
s1-osk-091-01+01_2a	ach	0.0681208636	0.2283709448	0.1837522472	0.166981292	0.0043161524
s1-osk-092-01+01_2a	ach	0.320622959	0.168104419	-0.004683845	0.036326225	0.1963835065
s1-osk-094-01+01_1a	ach	0.09199941	0.0968496717	-0.1887556656	0.1067494742	0.1019088465
s1-osk-095-01+01_2a	ach	0.2593935581	0.5370606737	-0.0040024422	0.101057113	-0.2290193116
s1-osk-095-01+01_2b	ach	0.2884601747	-0.273382385	-0.0766670486	-0.3470793051	0.2120171204
s1-osk-098-01+02_2b	ach	0.1172218038	0.094899769	0.7022154516	0.6246248579	0.6381995508
s1-osk-100-02+01_1a	ach	-0.0160702568	-0.025151309	-0.032685085	-0.0155860076	-0.0065022045
s1-osk-106-01+01_1a	ach	0.0350336724	0.0310827611	0.037816429	0.01488782	0.0188419288
s1-osk-107-02+01_1a	ach	0.077999096	-0.02754441	0.1336141841	0.0338193564	0.1386427822
s1-osk-109-01+01_1a	ach	-0.4012709307	0.0610794491	-0.0741062492	0.2757403515	-0.2004990358
s1-osk-110-01+01_1a	ach	-0.0476854283	-0.198902942	-0.153744049	-0.1766668199	-0.0246665897
s1-osk-115-01+01_1a	ach	0.2221766502	-0.156152002	0.2223879725	-0.0603061255	0.3122218002
s1-osk-115-01+01_1b	ach	-0.0168349491	0.2016634395	0.1246929488	0.1808567534	-0.0384152603
s1-osk-116-01+01_1a	ach	0.0876236328	0.4036445544	0.2026690007	0.2769389186	-0.0557248807
s1-osk-117-01+01_1a	ach	0.09183085	0.1081284267	0.0810524515	0.1052349653	0.0889279862
s1-osk-130-01+01_1a	ach	0.3227131654	0.1566440881	0.3078461033	-0.1541244039	0.0213625115
s1-osk-132-01+01_2a	ach	0.3179598067	0.156394281	0.3241389874	0.189835351	0.348604
s1-osk-134-02+01_1b	ach	0.1459874132	-0.292933541	0.4446124486	-0.1164330092	0.320179116

s1-osk-134-03+01_1a	ach	-0.1803900471	0.0869334107	-0.0159980559	0.069827587	-0.1969887787
s1-osk-136-01+01_2a	ach	0.1032606238	0.0956094519	0.058338988	-0.020638775	-0.0129133717
s1-osk-143-01+01_2b	ach	-0.2431942296	0.1400224923	-0.2209378338	0.2461074737	-0.13698322
s1-osk-145-01+01_1a	ach	0.0027311797	-0.007610749	0.0829157087	0.0577140499	0.0680151681
s1-osk-145-02+01_1a	ach	0.0824081675	0.0390216936	-0.3411715495	-0.417789466	-0.3811950128
s1-osk-147-01+01_1a	ach	0.0184282593	0.270301303	-0.0623101972	0.0186620386	-0.2355848131
s1-osk-147-01+01_2a	ach	0.0300410321	0.0260639584	-0.1579837482	-0.2322141846	-0.2284451365
s1-osk-147-01+01_2b	ach	0.0122370772	-0.075663037	0.006916238	0.1523502339	0.236998187
s1-osk-150-01+01_1a	ach	0.1258822168	0.1283880111	-0.2273051509	0.1745463078	0.1720758486
s1-osk-150-01+01_1b	ach	0.080876898	0.0291666643	0.1113098614	-0.145892649	-0.0947768478
s1-osk-150-01+01_1c	ach	-0.2692130794	-0.002117368	-0.0956885843	-0.0492843401	-0.3124174938
s1-osk-155-01+01_1a	ach	-0.188611366	-0.204928683	-0.0557296615	-0.249817555	-0.2338357058
s1-osk-155-01+01_1b	ach	0.0942111731	0.213324836	0.1865659097	0.1809368184	0.0607155427
s1-osk-155-02+01_1a	ach	0.0760173288	0.0588044824	-0.1136893927	-0.0668921113	-0.0496594101
s1-osk-155-02+01_1b	ach	0.3844180499	0.0854084887	0.1568592824	0.091968781	0.3900388562
s1-osk-157-01+01_2a	ach	0.3179531056	0.1530799557	0.0447539019	0.0556185423	0.226741407
s1-osk-159-01+01_1a	ach	-0.0227088228	-0.013046996	0.1764947817	0.072973614	0.0633536046
s1-osk-159-01+01_1b	ach	0.1308641249	-0.09618827	-0.4579958146	-0.6326185345	-0.4758953513
s1-osk-160-01+01_1a	ach	0.0728186688	-0.106202764	-0.0606267108	-0.141687946	0.0368881345
s1-osk-160-01+01_1b	ach	0.0926664956	0.319524146	-0.0577034392	0.0173837632	-0.2172789275
s1-osk-160-02+01_1a	ach	0.0579585304	0.0214088893	0.2547258328	0.2432194163	0.2773459131
s1-osk-162-01+01_2a	ach	0.0876839641	0.1043952135	-0.2090311345	-0.1991034428	-0.2152462784
s1-osk-164-01+01_2b	ach	-0.1674812308	-0.048006043	0.3011036556	0.1272826763	0.006945587
s1-osk-165-01+01_2a	ach	-0.1922843002	-0.063283401	0.3604872709	0.4407204586	0.3290696543
s1-osk-166-01+01_1a	ach	0.2751056472	0.3661803091	0.3253822077	0.0766789531	-0.0247907138
s1-osk-166-01+01_2a	ach	-0.0613989446	0.0340714603	0.1885393006	0.2586895555	0.1675477751
s1-osk-167-01+01_1a	ach	-0.0359034667	0.1077944447	-0.2467980811	-0.0006334817	-0.1437643619
s1-osk-167-01+01_2a	ach	-0.0521084321	0.2824687773	-0.5127192161	-0.44339122	-0.6745048229
s1-osk-168-01+01_2a	ach	0.0700661651	0.0217044427	0.0191043096	-0.0240451942	0.024418625
s1-osk-169-01+01_1a	ach	0.0130576394	-0.005959473	-0.0563310669	0.0288361216	0.0478255294
s1-osk-169-01+01_2a	ach	0.0905087646	0.0348425351	0.0850834638	0.3421900117	0.3905690804
s1-osk-177-01+01_2a	ach	-0.0802273663	-0.203531573	0.2342273423	0.1214569452	0.2431067436
s1-osk-180-01+01_1a	ach	0.1265158972	0.1638582525	-0.1316743728	-0.13789877	-0.1751108293
s1-osk-180-01+01_2a	ach	-0.069610597	-0.020367646	0.0111845373	0.0533038964	0.0040015475
s1-osk-182-01+01_1a	ach	-0.0482472053	0.0217719973	0.1522599054	0.1625482888	0.0936675182
s1-osk-182-01+01_2a	ach	-0.1796957182	0.1061139738	-0.0664019477	-0.1450145102	-0.4088480238
s1-osk-184-01+01_2a	ach	0.031001068	0.0360225713	-0.2401351041	-0.3243263031	-0.3288173079

s1-osk-184-01+01_2b	ach	-0.0525152114	0.3088932816	-0.0786948741	0.0002199357	-0.35544732
s1-osk-185-01+01_2a	ach	-0.0525913194	0.164975409	0.1299380409	0.0378208157	-0.1793374754
s1-osk-187-01+01_1a	ach	-0.1328995598	0.0430441502	0.0764459358	-0.0738988926	-0.245665843
s1-osk-187-01+01_1b	ach	0.303628068	0.1057074808	-0.0299358503	-0.0011466263	0.2033847053
s1-osk-188-01+01_1a	ach	0.2232665474	0.0978814942	-0.1589055493	-0.4561883944	-0.3483739109
s1-osk-188-01+01_1b	ach	0.1060920759	0.1406738093	-0.012885462	0.0071063114	-0.0280063389
s1-osk-189-01+01_1a	ach	0.1087971037	0.1949740166	-0.1798560467	-0.0820239344	-0.1688491141
s1-osk-189-01+01_1b	ach	0.3955548046	0.1716554646	0.6155946646	0.564636242	0.7087211066
s1-osk-191-01+01_1d	ach	0.1762795015	0.0713714838	-0.0592462974	-0.1951065502	-0.0907428448
s1-osk-191-01+01_2a	ach	-0.1506806506	-0.108038458	-0.2874021594	-0.3048167416	-0.3436242467
s1-osk-192-01+01_1a	ach	0.1735860344	0.1786782164	0.2645991135	0.1430913944	0.1379399444
s1-osk-193-01+01_1c	ach	0.0658461468	0.0223209856	-0.2412452454	-0.3426966599	-0.30364322
s1-osk-194-01+01_1a	ach	0.3676564627	0.0505467262	0.4212925338	0.1032633498	0.4126106724
s1-osk-195-01+01_1a	ach	0.1025915064	-0.026129566	0.1305271999	0.0084562649	0.1366848177
s1-osk-195-01+01_1b	ach	0.0389846401	0.0528850188	-0.0300471983	-0.021242878	-0.0351615702
s1-osk-196-01+01_1a	ach	0.0622994109	0.0644537098	-0.262797071	-0.2117749801	-0.2138400115
s1-osk-198-01+01_2a	ach	0.0057142114	-0.102357949	-0.18171844	0.0243203596	0.1319826519
s1-osk-199-01+01_1a	ach	0.0198636206	-0.00414533	-0.0107654513	0.0876544251	0.1114269214
s1-osk-199-01+01_1c	ach	0.0952229627	0.0027909098	0.0017361922	-0.0405951611	0.0520568476
s1-osk-199-02+01_1a	ach	-0.0021719667	0.1004658825	0.085278262	0.060918109	-0.0419596442
s1-osk-200-01+01_1a	ach	0.0245171373	0.1019830826	0.1012939413	0.0612003585	-0.0165383669
s1-osk-200-01+02_1a	ach	0.0029937284	0.130638374	-0.0468193336	0.0115467238	-0.1163193702
s2-osk-082-01+01_2a	ach	-0.0817960578	0.0463271587	0.0954158997	0.1245043713	-0.0031857983
s2-osk-083-01+01_2a	ach	0.1977631794	0.1775377608	0.0024593782	-0.0771109571	-0.0562404797
s2-osk-084-01+01_1a	ach	0.0224760089	0.0688599396	0.0511518316	0.0620195864	0.0156087276
s2-osk-086-01+01_1a	ach	-0.0076209344	0.1049329515	-0.0132623879	0.0214925635	-0.0911918098
s2-osk-088-01+01_2a	ach	0.0343291775	0.1994072646	0.019959995	-0.0108127914	-0.176711112
